# A chromosome-scale genome assembly of turmeric provides insights into curcumin biosynthesis and tuber formation mechanism

**DOI:** 10.3389/fpls.2022.1003835

**Published:** 2022-09-26

**Authors:** Yanpeng Yin, Xiaofang Xie, Luojing Zhou, Xianmei Yin, Shuai Guo, Xianjian Zhou, Qingmiao Li, Xiaodong Shi, Cheng Peng, Jihai Gao

**Affiliations:** ^1^ State Key Laboratory of Southwestern Chinese Medicine Resources, Chengdu University of Traditional Chinese Medicine, Chengdu, China; ^2^ Key Laboratory of Bio-Resource and Eco-Environment of Ministry of Education, College of Life Sciences, Sichuan University, Chengdu, China; ^3^ Sichuan Provincial Key Laboratory of Quality and Innovation Research of Chinese Materia Medica, Sichuan Academy of Traditional Chinese Medicine Sciences, Chengdu, China; ^4^ School of Food and Biological Engineering, Chengdu University, Chengdu, China

**Keywords:** *Curcuma longa*, curcumin biosynthesis, spices, tuber formation, genome assembly

## Abstract

*Curcuma longa*, known as the ‘golden spice’ and ‘life spice’, is one of the most commonly utilized spices in the world and also has medicinal, cosmetic, dye and flavoring values. Herein, we present the chromosomal-level genome for turmeric to explore the differences between tubers and rhizomes in the regulation of curcumin biosynthesis and the mechanism of tuber formation. We assembled the turmeric genome into 21 pseudochromosomes using Pacbio long reads complemented with Hi-C technologies, which has a total length of 1.11 Gb with scaffold N50 of 50.12 Mb and contains 49,612 protein−coding genes. Genomic evolutionary analysis indicated that turmeric and ginger have shared a recent WGD event. Contraction analysis of gene families showed possible roles for transcription factors, phytohormone signaling, and plant-pathogen interactions associated genes in adaptation to harsh environments. Transcriptomic data from tubers at different developmental stages indicated that candidate genes related to phytohormone signaling and carbohydrate metabolic responses may be associated with the induction of tuber formation. The difference in curcumin content between rhizomes and tubers reflected the remodeling of secondary metabolites under environmental stress, which was associated with plant defense in response to abiotic stresses. Overall, the availability of the *C. longa* genome provides insight into tuber formation and curcumin biosynthesis in turmeric as well as facilitating the understanding of other *Curcuma* species.

## Introduction

**Figure 1:**
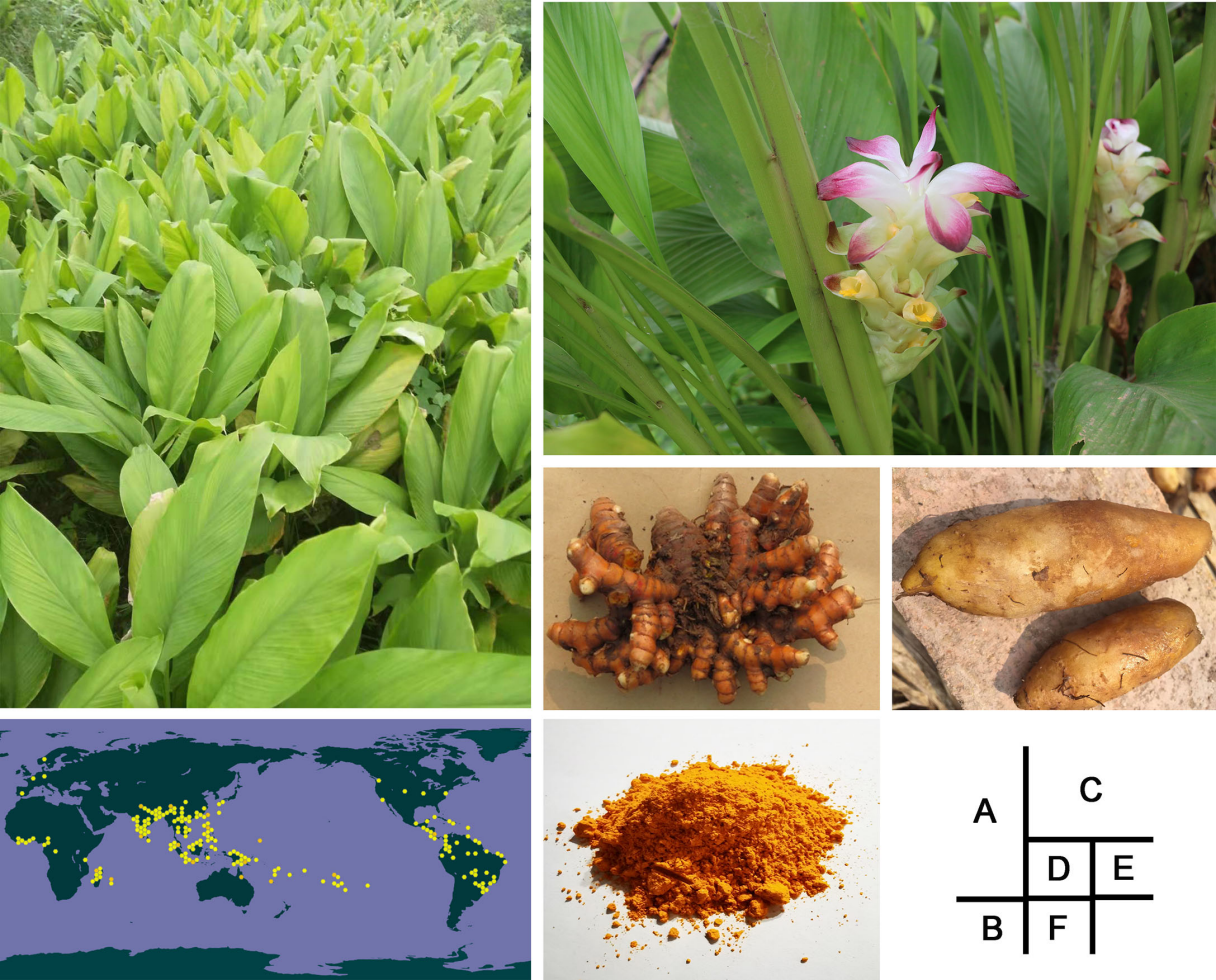
Morphological traits of Curcuma longa. **(A)** image of C *longa*. **(B)** global distribution range of C *longa*, which was obtained from the GBIF website (www.gbif.org). **(C)** a photographs of C *longa* flowers, **(D)** rhizomes, **(E)** tubers, **(F)** turmeric powder.

The genus *Curcuma*, comprised of 70 species, the largest genus in the Zingiberaceae family, is generally distributed in tropical and subtropical regions, including China, India, Thailand, Indonesia and Malaysia, with a few species occurring in Australia and the South Pacific (**Figure 1B**) (Kocaadam and Şanlier, 2017; Zhang et al., 2018). It has many cultivars as its highly variable morphology and the wide range of chromosome numbers in the genus, with diploid, triploid and tetraploid plants (Leong-Skornicková et al., 2007; Záveská et al., 2016). *Curcuma longa* belongs to the triploid species (2n=3x=63), a rhizomatous perennial herb whose rhizome is used as one of the most common sources of spices in the world (**Figure 1**). Usually, *C. longa*, also referred as “turmeric”, was said to be widely used for treat a variety of diseases in Asian countries for at least 2,500 years (Gupta et al., 2013). Due to its special flavor, turmeric has been widely used as a flavouring agent, cosmetic, textile dye and so on. Several secondary metabolites have been reported from turmeric roots, including flavonoids (mainly curcuminoids), phenylpropanoids and terpenoids (Sun et al., 2017), which play important roles in plant growth and development, as well as stress resistance (Kroymann, 2011; Geng et al., 2020; Gu et al., 2020). Curcumin, the main medicinal ingredient and natural golden spice distributed the rhizomes of turmeric, belongs to curcuminoid, an extremely rare diketone colored substance in nature, mainly including curcumin, demethoxycurcumin, bisdemethoxycurcumin and so on. According to the modern pharmacological research, curcumin has anti-oxidant, anti-inflammatory, analgesic, anti-tumor, anti-diabetic effects (Prasad et al., 2014; Shehzad et al., 2017). In addition, as one of the natural colors with a high safety, curcumin has been used to add color and flavor to foods (Sharma et al., 2005; Prasad et al., 2014).

In China, the rhizomes and tubers of turmeric are used as two different herbal medicines (named as “Jiang Huang” and “Huang Si Yu Jin”, respectively), which have different clinical uses and differ greatly in their curcuminoid content (Sun et al., 2018; Liao et al., 2020). Turmeric tuber is rarely produced in practice, but it is undoubtedly a valuable part. The formation of tubers is commonly caused by environmental and endogenous factors, e.g., moisture, temperature, light, high sucrose and hormonal changes (Ravi et al., 2009; Aksenova et al., 2012). The transition from rhizomes terminal enlargement to tubers of turmeric is a complex biological process involving many changes of genes. Therefore, increasing our understanding of genetic factors in medicinal plant roots may be important for our understanding of how medicinal compounds, such as curcumin, are biosynthesized and the regulatory relationships between survival and secondary metabolites under abiotic stresses (Jogawat et al., 2021). To date, limited dataset resources restrict further elucidation of the genetics underlying of these interesting traits. Despite the great value of turmeric tubers, there are deficiencies in the mechanism of formation, biosynthesis of active ingredients and other related molecular biological basis.

High-quality genomes enable to understand the basic genes involved in curcumin biosynthesis and the traits for tuber formation. Here, we present the chromosome-level genome of *C. longa* in Zingiberaceae family, with PacBio, Hi-C and Illumina sequencing technology, and combined transcriptomic data to identify a number of candidate genes for curcumin biosynthetic pathway. Our data derived from developing rhizomes and tubers may be useful in analyzing transcriptomic changes associated with tuber development. The reference genome of *C. longa* provides insights into the biosynthesis of curcumin and development of applications in Zingiberaceae.

## Result

### Genome sequencing and assembly

**Figure 2:**
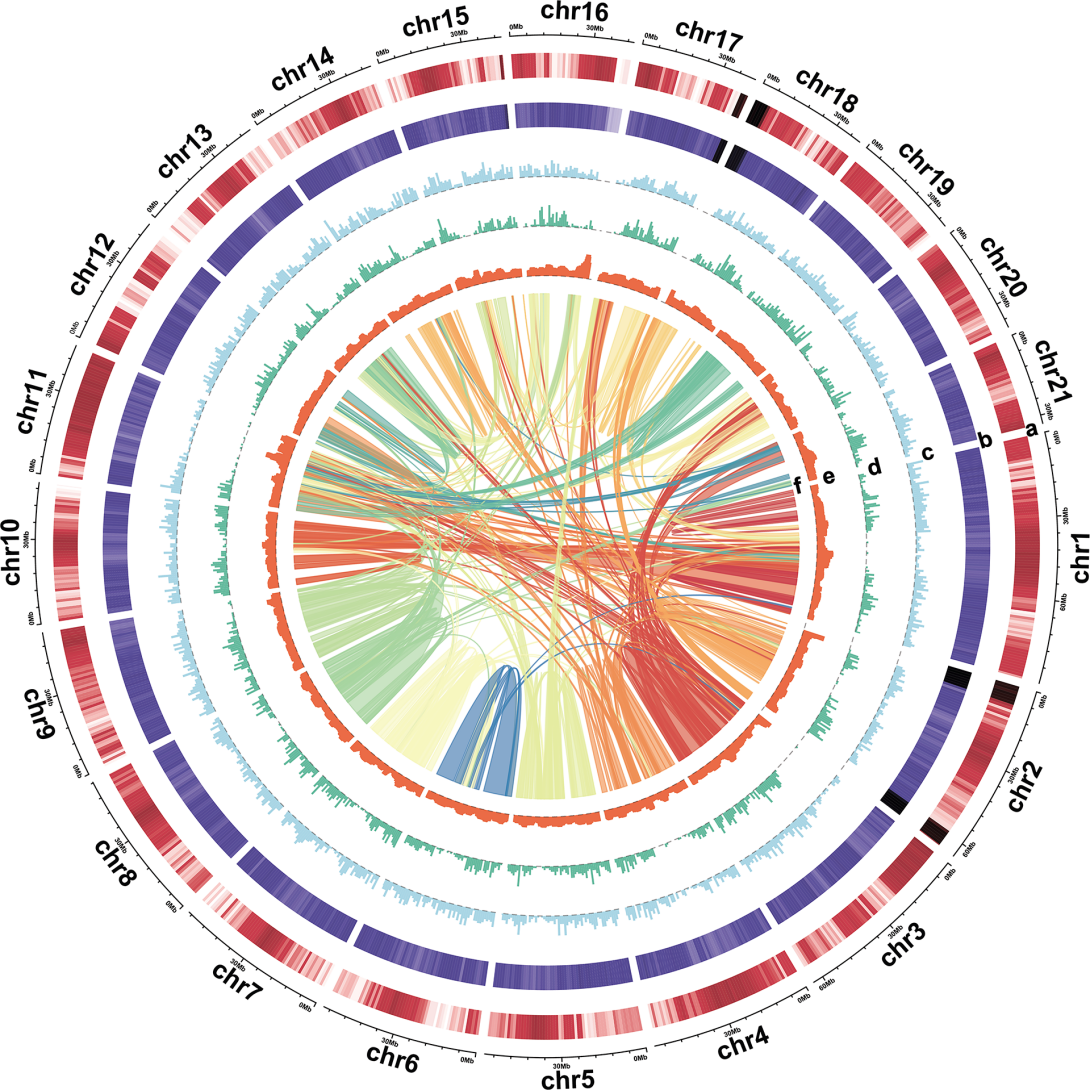
Characterization of the turmeric genome. Tracks: **(A)** Gene density, **(B)** GC content, **(C)** Copia density, **(D)** Gypsy density, **(E)** LTR assembly index (LAI), and **(F)** synteny block.

A triploid turmeric (2n=3x=63) plant was selected for genome sequencing and assembly. In total, 89X Illumina short reads, 202X PacBio long reads, and 96X Hi-C reads were generated for turmeric (**Table S1**). *C. longa* genome size was estimated to be approximately 1.19 Gb, and the heterozygosity rate was 3.53% based on the *K*-mer analysis method using Illumina short reads (**Figure S1**). PacBio long reads were used for the initial assembly, with an average read length of 25.6 kb (**Figure S2**), and redundant sequences from heterozygous genomic regions were filtered out using purge_haplotigs, then the contig sequences were further polished using Illumina short reads. The de-redundancy assembled genome size was 1.11 Gb with a contig N50 of 2.24 Mb, and the GC content was 40.18% (**Table 1** and **Table S2**). Then the assembly was enhanced and adjusted by 106.73 Gb Hi-C reads to provide a chromosome-level assembly, resulting in a 1.11 Gb genome with a scaffold with N50 of 50.12 Mb (**Table 1** and **Table S2**). 96.25% of the assembled sequences were anchored onto 21 pseudochromosomes (**Figure 2**; **Figure S3** and **Table S3**). Based on this assembly result, we evaluated the genome completeness of *C. longa* using BUCSO with the embryophyte_odb10 database. The genome assembly completeness reached 95.2%, while 1.1% and 3.7% were partially present or missing, respectively (**Table S4**). The final LTR assembly index (LAI) value for turmeric was 13.56, which meets the reference quality (10 ≤ LAI < 20) (Ou et al., 2018)and is higher than that of a previous assembled turmeric (10.26) (Chakraborty et al., 2021), indicating that our assembly was high integrity.

**Table 1:** The major characteristics of *C. longa* (2n=3x=63) genome.

Sequencing platform	Illumina, PacBio, Hi-C
**Assembly**	
Assembly level	Chromosomes
Total length	1.11Gb
Number of contigs	680
Contig N50	2.34Mb
Number of scaffolds	101
Scaffold N50	50.12Mb
GC content	40.18%
Complete BUSCOs	95.2%
Complete and single-copy BUSCOs	70.4%
Complete and duplicated BUSCOs	24.8%
Fragmented BUSCOs	1.1%
Missing BUSCOs	3.7%
Average LAI	13.56
**Annotation**	
Repeat sequences	69.99%
Number of protein-coding genes	49,612
Number of non-coding RNAs	3,468

### Genome annotation

We annotated the repetitive elements of the turmeric genome through homology-based, methods and *ab-initio* prediction, and 69.99% of the assembly was determined to be composed of repetitive elements. Among them, retrotransposons accounted for 60.98%, and DNA transposons accounted for 8.40%. More than 50% of the *C. longa* genome consisted of long terminal repeat retrotransposons (LTR-RTs), The percentages of Gypsy and Copia were 21.40% and 31.09% (**Table S5**). For protein-coding gene annotation, we used a combination of three methods: homology-based, *ab initio*, and RNA-Seq prediction methods. Overall, 60,686 protein-coding genes were predicted in our *C. longa* assembly, among 49,612 protein-coding genes were assigned functions in the five databases (NR, TrEMBL, Swissprot, KOG and KEGG) (**Table S5**). Moreover, the genes annotated as ncRNAs included 354 microRNAs, 1,829 tRNAs, and 1,285 rRNAs (**Table S7**).

### Comparative genomics analysis

**Figure 3:**
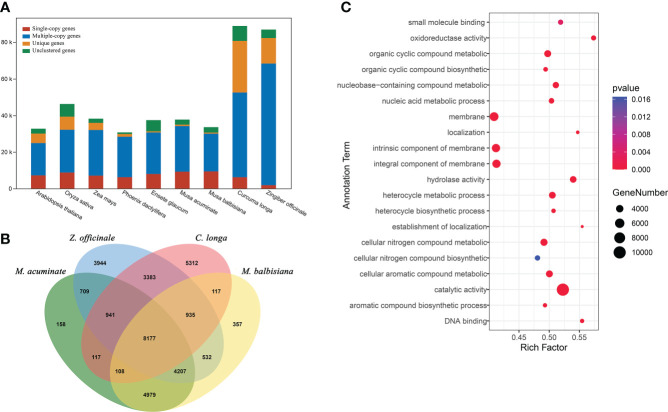
Comparative genomic analysis of *C. longa*. **(A)** Orthologous genes found in different plant species. **(B)** Venn diagram of the number of shared gene families within *C. longa*, *Z. officinale*, *M. balbisiana* and *M. acuminate*. The number in the Venn diagram is the number of gene families. **(C)** GO enrichment analysis of gene families specific to turmeric.

To understand the evolutionary dynamics of the *C. longa* genome, we collected genome sequences of representative species (including *Arabidopsis thaliana*, *Oryza sativa*, *Phoenix dactylifera*, *Zea mays*, *Ensete glaucum*, *Musa acuminate*, *Musa balbisiana* and *Zingiber officinale*), and performed a comparative genomic analysis with the genome sequence of *C. longa*. In the *C. longa* genome, there were 52,499 candidate orthologs gene families, 5,222 of which were specific to *C. longa* (**Figure 3A** and **Table S8**). In addition, we focused on the gene families of species closely related to turmeric, with a total of 8,177 gene families were shared by Zingiberales (*C. longa*, *Z. officinale*, *M. balbisiana* and *M. acuminate*) (**Figure 3B**). Gene Ontology (GO) annotation showed that these turmeric genome-specific genes were enriched mainly in catalytic activity (GO:0003824), oxidoreductase activity (GO:0016491), hydrolase activity (GO:0016787), membrane (GO:0016020), integral component of membrane (GO:0016021), cellular nitrogen compound metabolic process (GO:0034641), cellular aromatic compound metabolic process (GO:0006725) and organic cyclic compound metabolic process (GO:1901360) (**Figure 3C** and **Table S9**). Oxidoreductase activity may have developed to help plants scavenge ROS and eliminate oxidative damage (Lv et al., 2020), and antioxidant defense is fundamentally connected to redox signaling, cellular communication, and acclimation (Luthje et al., 1997; Liebthal et al., 2018). In total, unique gene families in turmeric were mainly related to defense functions, cell membrane composition, and cellular metabolic processes.

### Genome evolution

**Figure 4:**
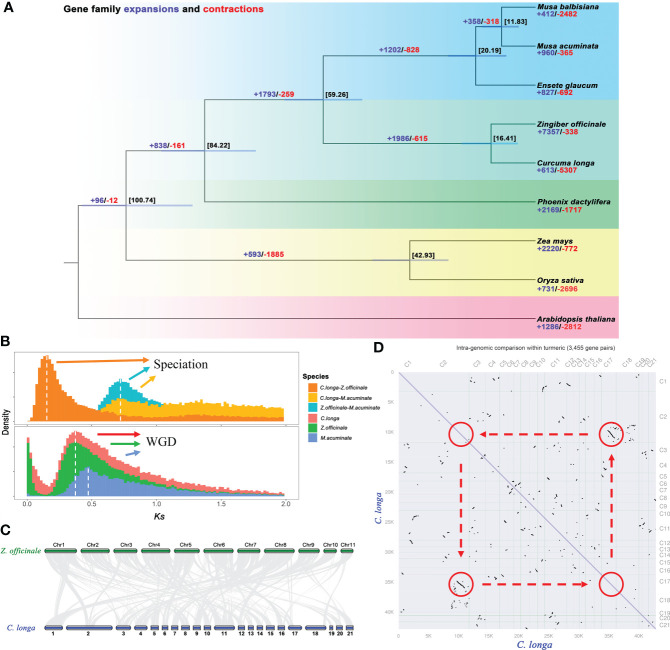
Genome comparison and evolution analysis. **(A)** Phylogenetic tree of *C. longa* and eight other species based on single-copy protein sequences, and divergence times (Mya) are indicated by the numbers beside the branch nodes. The numbers of contracted gene families are in blue, and those of expanded gene families are in red. **(B)** Distribution of the synonymous substitution (Ks) rates for *C. longa*, *Z. officinale*, and *M. acuminate* genome, peaks in the intraspecies Ks distribution indicate whole-genome polyploidization events, and peaks in the interspecies Ks distribution indicate speciation events. **(C)** Synteny conservation between *C. longa* and *Z. officinale* genomes. **(D)** Dot plots of paralogs in the *C. longa* genome.

The species of Zingiberaceae are a controversial and complex (taxonomic status) flowering family, and more genetic data are needed to confirm their relationships (Kress et al., 2002; Liang et al., 2020). A phylogenetic tree was constructed based on 119 single-copy orthologous genes in *C. longa* and eight other species. The phylogenetic tree revealed that *C. longa* is strongly related to *Z. officinale*, these are both from the Zingiberaceae lineage, and diverged from their common ancestor at ~16 Mya. Further estimated the times of divergence among these plants, i.e., the Musaceae (*M. acuminate*, *M. balbisiana* and *E. glaucum*) separated from the Zingiberaceae (*C. longa* and *Z. officinale*) at approximately 59 Mya (**Figure 4A**). Expansion and contraction of gene families are significant features of species selective evolution. We detected 613 significant expansion and 5307 significant contraction gene families in turmeric genomes (**Figure 4A**). The expanded gene families were enriched in secondary metabolic processes and carbohydrate metabolism, including phenylalanine, polyketides, terpenoids, butanoate and propanoate (**Figure S4**), while contracted gene families were mainly related to transcription factors, plant hormone signal transduction and plant-pathogen interaction (**Figure S5**). Selective expansion or contraction of these gene families that have been preserved during evolution suggests that turmeric is adaptively altered to its environment. In addition, a significant expansion of 7,357 gene families was detected in the ginger genome compared to turmeric, which may be one of the reasons for the large difference in genome size between the both (Cheng et al., 2021).

Whole-genome duplications events were one of most important drivers of genome evolution (Van De Peer et al., 2009a; Van De Peer et al., 2009b), and we used synonymous substitution rates (Ks) values to estimate the timing of large-scale duplication (Blanc and Wolfe, 2004). A peak of duplication with Ks approximately 0.35 was found in *Z. officinale* and *C. longa* genome, suggesting that a recent whole-genome duplication event occurred in the Zingiberaceae clade (**Figure 4B**) (Cheng et al., 2021; Li et al., 2021). The dot plot (**Figure 4D**) also illustrates the paralogs inherited from the recent WGD in the turmeric genome (2-2 diagonal relationships). The Musa lineage also experienced an ancient WGD event (Ks= ~ 0.45) (D’hont et al., 2012; Wu et al., 2016) (**Figure 4B**). Comparison of interspecies between the orthologs gene pairs in *C. longa vs. M. acuminate*, and *Z. officinale vs. M. acuminate*, indicated that the divergence of Zingiberaceae and Musaceae (Ks= ~ 0.7) before the recent WGD event (Ks= ~ 0.3-0.4) (**Figure 4B**). A colinear maps were constructed by comparing the turmeric genome with the ginger genome, we found a total of 564 syntenic blocks (21,829 pairs of collinear genes) between turmeric and ginger (**Figure 4C** and **Figure S6**). Synthetic depth analysis showed a 2:2 ratio of genes observed in the comparison of turmeric and ginger genomes (**Figure S7**).

### Genes involved in curcumin biosynthesis

Turmeric has a distinctive spicy aroma and it is well known as a spice. However, only a few species in the Zingiberaceae family have the unique flavor of turmeric, and the compounds of turmeric rhizomes and tubers were analyzed by UPLC-MS/MS. Phenols and terpenoids are the two main classes of compounds that were detected, including curcumin and its derivatives. Curcumin is the recognized one of the main ingredient in *C. longa* with a variety of biological activities and pharmacological effects (Dosoky et al., 2019). The accumulation of large amounts of curcumin in the rhizome is remarkable distinctive feature of turmeric (Fabianowska-Majewska et al., 2021). Then we analyzed the genes in the curcumin biosynthetic pathways.

**Figure 5:**
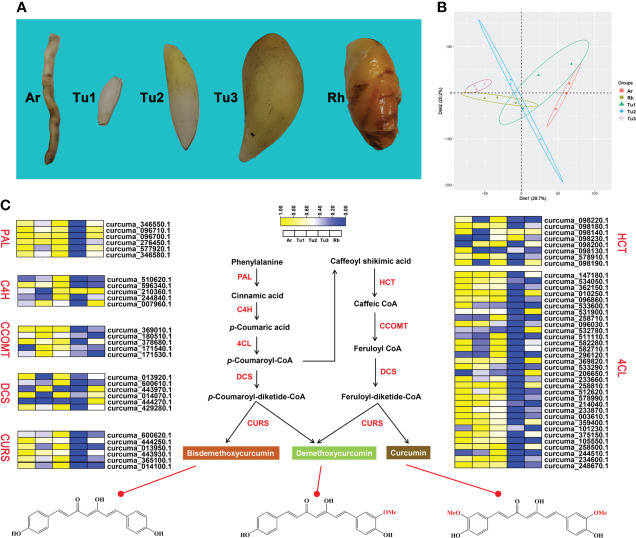
Curcumin biosynthesis pathway in turmeric. **(A)** Phenotypic comparison of five root developmental stages of turmeric. **(B)** Principal component analysis (PCA) of five turmeric tissues based on the transcriptomic data. Three biological replicates were performed for each developmental stage. **(C)** Relative expression profiles (yellow-blue scale) of genes encoding enzymes possibly involved in curcumin biosynthesis (heat map). The enzymes involved at each step are shown in red.

**Figure 6:**
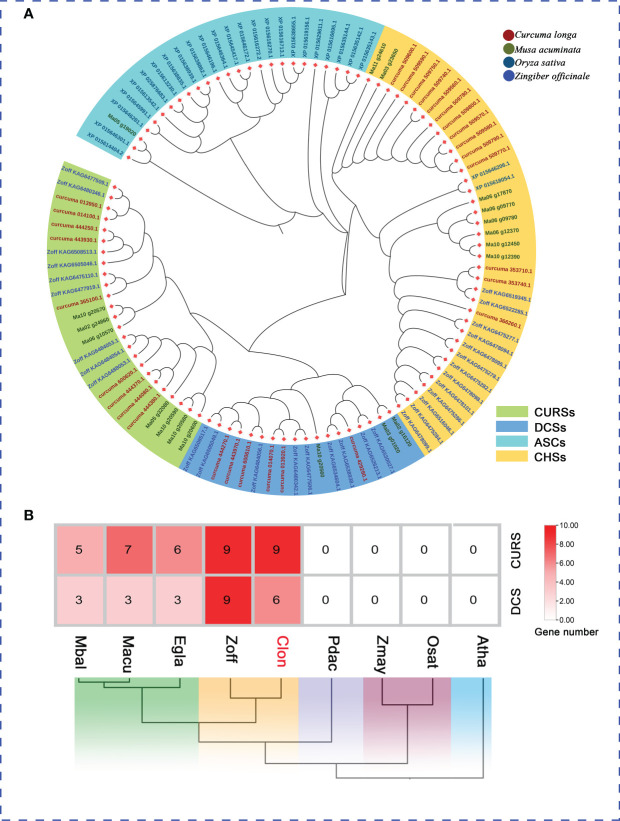
The PKS Genes Clusters Identified in the *C. longa* Genome. **(A)** Phylogenetic analysis of PKS genes from *C. longa*, *Z. officinale*, *M. acuminate* and *O. sative*. Different colored boxes represent the classification of PKSs. **(B)** Distribution pattern of the expanded gene families in curcumin biosynthesis pathway and comparison with other species.

Previous studies identified some key enzymes in the curcumin biosynthetic pathway and confirmed the phenylpropanoid pathway are involved in the biosynthesis of curcumin (Ramirez-Ahumada et al., 2006). In this study, we identified genes involved in this pathway based on the genome-wide level of turmeric, and evaluated and compared their expression levels in rhizomes (Rh), adventitious roots (Ar), and tubers at different developmental stages (Tu1-Tu3) (**Figure 5A**). Transcriptomic data showed that Rh, Ar and Tu1-3 had different gene expression profiles (**Figure 5B**), indicating the presence of different regulatory events in the roots at different developmental stages. We identified 68 candidate genes associated with the curcumin biosynthesis in five tissues, and these genes were mainly encoding seven key enzymes, e.g., phenylalanine ammonia-lyase (PAL), cinnamate 4-hydroxylase (C4H), 4-coumarate-CoA ligase (4CL), shikimate O-hydroxycinnamoyl transferase (HCT), caffeoyl-CoA O-methyltransferase (CCOMT), diketidyl-CoA synthase (DCS) and curcumin synthase (CURS) (**Figure 5C**). DCS and CURS are the key enzymes responsible for biosynthesis of curcumin compounds, and they are involved in significantly expanded genes in turmeric same as in ginger (**Figure 6B**). DCS synthesizes feruloyl diketide-CoA and coumaroyl diketide-CoA, and CURS then converts the diketide-CoA esters to curcuminoid (Katsuyama et al., 2009). DCS and CURS had higher mRNA levels in rhizomes compared with tubers, whereas the expression levels of both increased from Tu1 to Tu2 stage, finally decreased from Tu2 to Tu3 stage according to the three stages of tubers development (**Figure 5C**). These two genes had similar expression patterns in adventitious roots, and the expression levels were greatly higher compared with that in rhizomes and tubers. As the result of UPLC-MS/MS analyses, curcuminoids were more abundant in the rhizomes than in the tubers (**Table 2** and **Figure S8**). This result was consistent with the correlation between gene expression and metabolite concentrations in plant secondary metabolite biosynthesis, where these related genes were often expressed coordinately (Li et al., 2015). In agricultural production, our investigation showed that the formation of turmeric tubers may be related to water-deficit conditions. A study showed that the expression levels of CURS and DCS were upregulated in turmeric under water-deficit conditions (Chintakovid et al., 2022), which was consistent with the expression pattern of the tuber formation stage (Tu1 to Tu2). In addition, the difference in curcumin content between rhizomes and tubers reflected the remodeling of secondary metabolites under environmental stress, which was related to plant defense in response to abiotic stresses (Obata and Fernie, 2012; Zhu, 2016).

**Table 2:** The *m/z* value and fold change of curcuminoid in turmeric different tissues (tubers and rhizomes).

m/z(Observed)	m/z(Theoretical)	Formula	Chemical component	Fold Change(rhizome/tuber)
368.1255	368.1260	C_21_H_20_O_6_	Curcumin	1.24
338.1149	338.1154	C_20_H_18_O_5_	Demethoxycurcumin	2.36
308.1043	308.1049	C_19_H_16_O_4_	Bisdemethoxycurcumin	3.22

### Identification of polyketide synthases (PKS) gene family

The plant type III polyketide synthase (PKS) gene family, which catalyzes and synthesizes a variety of different structural and biologically active plant secondary metabolites, can provide unique polyketide backbones for various metabolic pathways (e.g., curcuminoids, flavonoids, stilbenes, diarylheptanoids and quinolones) associated with various functions of plant growth, development and defense (Austin and Noel, 2003; Dao et al., 2011; Yu et al., 2012a; Pothiraj et al., 2021). To further validate the subtype designation of *C. longa* PKSs, three species (*Z. officinale*, *M. acuminate*, and *O. sativa*) were selected for comparative analysis of PKS gene families. A total of 30 putative PKS proteins in the *C. longa* genome were annotated according to the conserved domains (PF00195 and PF02797) using HMMER. We constructed a phylogenetic tree by aligning the PKS proteins among *C. longa*, *Z. officinale*, *M. acuminate* and *O. sativa* to confirm the classification of PKS family proteins in turmeric. The 30 turmeric PKS genes were clustered into four groups, the known chalcone synthases (CHSs), DCSs, CURSs and anther specific CHS (ASCs) (Pothiraj et al., 2021) (**Figure 6A**). ASCs were specifically expressed in pollen development and comprise a monophyletic clade (Ageez et al., 2005; Jiang et al., 2008). CHSs, and DCSs along with CURSs were regarded as key enzymes for flavonoids, and curcuminoid biosynthesis, respectively. We further examined the genomic dataset to determine the copy number of genes involved in the biosynthesis pathways of curcumin, which revealed that the genes encoding CURS and DCS were expanded in the turmeric and ginger genomes (**Figure 6B**). The striking expansion of the DCS and CURS gene families in turmeric and ginger underpin the existence of curcuminoids mainly in Zingiberaceae. Our work identified the taxonomic and functional characteristics of the turmeric type III PKS genes, which provided a reference for systematic analysis of the functions of the PKS gene family and a basis for further studies on the biosynthesis of related secondary metabolites in Zingiberaceae.

### Analysis of gene expression patterns in turmeric tuber formation

**Figure 7:**
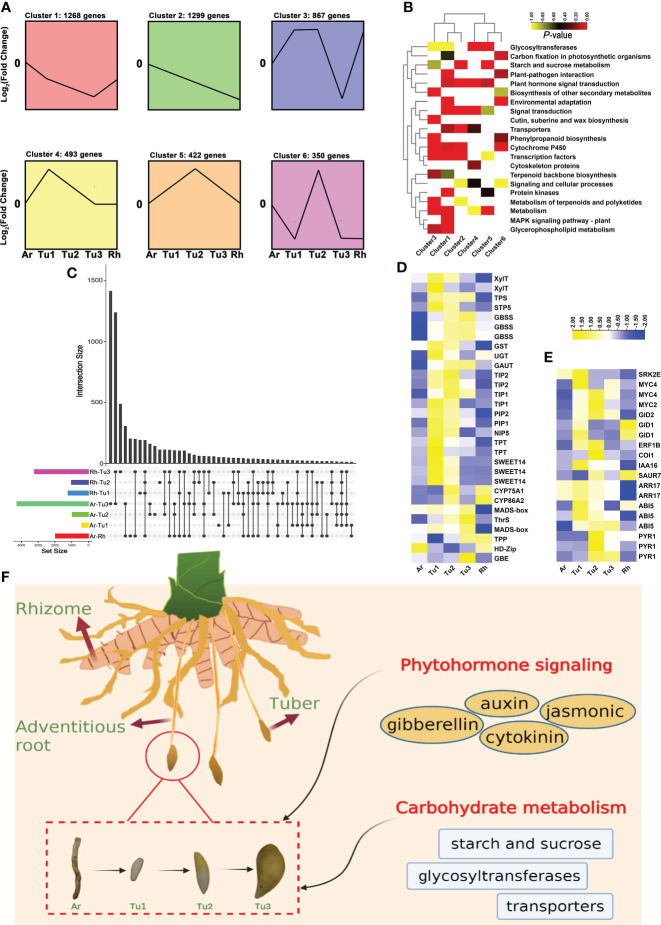
Expression patterns and enrichment analysis of DEGs in developing turmeric tubers. **(A)** Patterns of gene expressions across five turmeric tissues (Ar (adventitious roots), Tu1-3 (tubers1-3) and Rh (rhizomes)) inferred by STEM analysis. **(B)** Heat maps of significantly enriched pathways from six clusters. The yellow and red colors indicate the P-value for significantly enriched pathways. **(C)** Intersection diagram showing the distribution of differentially expressed genes among five turmeric tissues based on the transcriptomic data. **(D)** Heat maps for differentially expressed genes related to Tu1-Tu2 and Tu2-Tu3 stage. **(E)** Heat maps for differentially expressed genes related to phytohormone signaling pathways. **(F)** Schematic representation of overall effects of phytohormone signaling, carbohydrate on turmeric tuber development.

To further increase our understanding of the tuber formation and development, we performed transcriptomic analysis in rhizomes (Rh), adventitious roots (Ar) and three developmental stages tubers (Tu1-Tu3) of turmeric. Using STEM (Short Time-series Expression Miner) software, 6,868 DEGs (differentially expressed genes) from five tissues of turmeric were clustered into six clusters (from cluster 1 to 6) based on the expression patterns of the genes (**Figure 7A**). In general, cluster 3, 5 and 6 had 1639 genes and they all displayed a trend of increasing expression from Tu1 to Tu2 phase and decreasing expression from Tu2 to Tu3 phase (**Figure 7A**). KEGG enrichment analysis showed that these genes were related to starch and sucrose metabolism, secondary metabolism such as phenylpropanoid, terpenoids and polyketides, cytochrome P450 and cutin, suberine and wax biosynthesis, corresponding to tuber expansion, accumulation of secondary metabolites and environmental adaptation (**Figure 7B**). Another, there were 3,060 genes in cluster 1, 2 and 4, which showed a progressive decreasing expression trend from Tu1 to Tu3 stages, enrichment analysis indicating these genes related to transcription factors and plant hormone signal transduction (**Figure 7B**). For example, the transcription factor HY5 (HYPOCOTYL5) coordinates the metabolism of carbon and nitrogen in response to light, and hence shoot and root growth (Chen et al., 2016). AP2 (APETALA2) acts primarily in the regulation of developmental programs (Licausi et al., 2013). IAAs (auxin/indole-3-acetic acid) and ARFs (auxin response factors) are the key transcription factors in regulating the expression of auxin-responsive genes (Li et al., 2006), including IAA6/10/15/17/27, ARF11/19 and so on.

We also analyzed key DEGs at different developmental stages (**Figure 7C**). For the DEGs that were up-regulated in the Ar *vs.* Tu1 stage and were mainly involved in the biosynthesis of cell wall, nutrient element transporters and metabolism, and these genes were enriched in pathways including ‘glycosyltransferases’, ‘transporters’, and ‘starch and sucrose metabolism’ (**Figure S9A** and **Table S10**). Among these DEGs, we identified two XylT (xylosyltransferase), GAUT (galacturonosyltransferase), UGT (glucuronosyltransferase), three GBSS (granule-bound starch synthase), TPS (trehalose phosphate synthase), three SWEET (sugars will eventually be exported transporters), STP5 (sugar transport protein 5), NIP5 (NOD26-like intrinsic proteins 5), two TIP1 (tonoplast intrinsic protein 1), two TIP2, PIP2 (plasmamembrane intrinsic proteins), two TPT (triose phosphate/phosphate translocator) and GST (glutathione S-transferase). By comparing the gene expression profiles of Tu1 to Tu2 stage, we found that DEGs with higher transcript abundances in Tu2 stage were enriched in pathways including ‘cytochrome P450’, ‘cutin, suberine and wax biosynthesis’, and ‘flavone and flavonol biosynthesis’ (**Figure S9B** and **Table S11**). DEGs with higher expression in the Tu2 to Tu3 stage were enriched in ‘starch and sucrose metabolism’, ‘vitamin B6 metabolism’ and ‘transcription factors’ (**Figure S9C** and **Table S12**). Several genes related to cuticle development (CYP86A2), flavone biosynthesis (CYP75A1), starch synthesis (TPP, GBE), threonine biosynthesis (ThrS), regulation of growth and development (HD-Zip, MADS-box) were identified (**Figure 7D**). In summary, we found that genes involved in tuber development were significantly enriched in the carbohydrate metabolism, and genes of the transporter pathway were significantly enriched in the early stages of tuber formation, which contributed to the synthesis of sucrose in the leaves and its conversion to starch after transport to the tuber. In addition, phytohormones are among the important signaling substances that regulate plant development, growth and reproduction. In turmeric, genes related to tuber development were found to be significantly enriched in the phytohormone signaling pathways (**Figure 7E**), e.g., auxin, gibberellin, and cytokinin (Abelenda and Prat, 2013; Kondhare et al., 2021). For the auxin pathway, ARF and IAA regulate each other to participate in tuber development. In addition, other phytohormone-related genes including the PYR1 (abscisic acid receptor 1) (Santiago et al., 2009), SRK2E (serine/threonine-protein kinase) (Kulik et al., 2011), ABI5 (abscisic acid insensitive 5) (Collin et al., 2021). in the abscisic acid pathway, GID 1/2 (gibberellin insensitive dwarf 1/2) (Yoshida et al., 2018)in gibberellin pathway, ARR17 (arabidopsis response regulator 17) (Chang et al., 2019) in the cytokinin pathway, and COI1 (coronatine-insensitive protein 1) (Yan et al., 2009), transcription factor MYC2/4 (Van Moerkercke et al., 2019) in the jasmonic acid pathway were also highly expressed in the tuber development stage. These results suggested that regulation of phytohormones and carbohydrate metabolism genes contribute to the development of tubers (**Figure 7F**).

## Discussion

Given the importance of turmeric for edible and medicinal purposes, the genome of turmeric further enriches biological information for functional gene mining, tuber formation and secondary metabolite biosynthesis. The heterozygosity of turmeric is as complex as previously reported for the ginger genome (Li et al., 2021). In addition, turmeric, a triploid species, has a heterozygosity of 3.53%, which was a great challenge for genome assembly. We applied PacBio sequencing to assemble the genome by SMARTdenovo, using purge_haplotigs to filter redundant sequences in heterozygous genomic regions, and combined with Hi-C technology to assemble large-scale scaffolds into pseudochromosomes. In our case, this turmeric genome has a higher quality of assembly and annotation compared to older versions of turmeric draft genomes (Chakraborty et al., 2021). The complex diversity of morphology, chromosome numbers and chromosome sizes in curcuma species indicates that these species have a unique evolutionary history in which polyploid events may play an important role (Leong-Skornicková et al., 2007). Polyploidy is more common in the curcuma and zingiber genus (Anamthawat-Jónsson and Umpunjun, 2020). We identified the polyploidy events in two sequenced Zingiberaceae species, which observed one recent WGD shared between turmeric and ginger. This WGD event occurred before the diverged form ginger, and contraction of multiple gene families were detected in the turmeric genome, which may be responsible for its adaptation to harsh environments. Turmeric is mainly distributed in tropical and subtropical areas, and its smaller chromosomes may be the result of habitat adaptation (Chen et al., 2013). Consistent with previous studies, the changes in chromosome size may be related to the climate of the habitat, which species with smaller chromosomes were mainly distributed in tropical and subtropical climates, while larger chromosomes were distributed in temperate climates (Stebbins, 1966). This result will facilitate the study of the evolution of species and the molecular mechanisms of their adaptation to the environment.

Turmeric is one of the most popular spices in the world and its rhizomes are the main source of its ingredients. Compared to the rhizomes, the tubers of *Curcuma* species such as *C. longa*, *C. kwangsiensis*, *C. phaeocaulis*, *C. wenyujing* also have important value, e.g., spices, medicines, cosmetics, dyes and flavourings (Dosoky and Setzer, 2018). Current research on tuber formation focuses on potatoes, sweet potatoes, cassava and other crops (Ding et al., 2020; Zierer et al., 2021), but lack of research on the tuber development and formation mechanism of medicinal plants such as turmeric, which is necessary to fully elucidate the functions of medicinal plant roots in regulating the synthesis and distribution of secondary metabolites. Here, using a high-quality assembled turmeric genome, together with transcriptomic data during the development stages of the root system, we propose a regulatory mechanism for tuber formation and infer the contributions of genes related to phytohormone signaling and carbohydrate metabolism.

Basal metabolism plays a key role in the tuber formation of turmeric, e.g., carbon metabolism and starch and sucrose metabolism (Kondhare et al., 2020; Yoon et al., 2021). Our RNA-seq analysis revealed numerous genes were significantly up-regulated expressed in Tu1-Tu3 stage involved in carbohydrate metabolism, such as XylT, GBSS and SWEET. XylT participates in the biosynthesis of plant cell wall xyloglucan, which is the abundant hemicellulosic component of the primary cell wall (Ehrlich et al.; Ding et al., 2020). A high correlation between starch and sucrose content and tuber size. GBSS is a key enzyme for starch biosynthesis, and its expression promotes starch accumulation (Fulton et al., 2002). Plants that use sucrose as photosynthetic products realize the transport and distribution of photosynthetic products between source and sink organs through long-distance transport, which involves the transmembrane transport of sucrose (Riesmeier et al., 1994). SWEET participates in important physiological processes of plant growth and development by regulating the transportation, distribution and storage of sugar in plants (Chen, 2014; Breia et al., 2021). In addition, a series of enzymes such as GAUT, UGT, STP5, aquaporin (NIP5, TIP1, TIP2 and PIP2), TPT and GST are also involved in the tuber growth and development, including suger transport (Kong et al., 2019), photosynthesis (Lee et al., 2017) and stress response (Gullner et al., 2018; Kurowska, 2020; Zhang et al., 2021), suggesting their important roles in this process. Futhermore, the DEGs of phytohormone signaling pathway were significantly enriched in the developmental stage of turmeric tuber, inferring that hormones play an important regulatory role in the induction and formation of tuber. The formation and development of plant tubers and other storage organs are the result of the coordinated regulation of a variety of endogenous hormones (Hartmann et al., 2011; Roumeliotis et al., 2012). Several genes related to the biosynthesis of cytokinin, ABA, and IAA were identified, e.g., PYR1, SRK2E, ABI5, ARR17. Among them, cytokinin affects tuber formation and expansion, ABA affects tuber expansion by regulating the transfer of carbohydrates to tubers, and IAA promotes tuber expansion (Kondhare et al., 2021). During tubers growth and development, sugar and hormone signaling are usually tightly coupled (Gibson, 2005; Woodward and Bartel, 2005; Eveland and Jackson, 2012). However, the interaction between these genes and how they systematically regulate the formation and expansion of turmeric tubers still need further investigation.

In general, turmeric cannot adapt to harsh environments, such as water shortage and decreasing temperature, it can lead to dormancy of turmeric rhizomes (Rezvanirad et al., 2016; Chintakovid et al., 2021). A previous study screened the environmental factors that affect turmeric yield, such as longitude, latitude, soil pH, annual sunshine, and annual precipitation, and found that annual precipitation was one of the main influencing factors (Wu et al., 2019). However, its tubers can exist as a drought-resistant organ. Higher plants have evolved morphologically and physiologically to cope with challenging environmental conditions, and many have developed belowground storage organs such as tubers (Kondhare et al., 2021). Therefore, based on the results of surveys in turmeric growing areas and our research, we inferred that the formation of turmeric tubers is closely related to the drought environment, and according the above transcriptomic data, we found that some genes that resist abiotic stress also play a role in the development of tubers. Our results showed that genes such as GST, NIP5, TIP and PIP2 were significantly expressed at the tuber development stage, which could explain the presence of turmeric tubers as drought-tolerant organs. Curcumin as a secondary metabolite also plays a key role in the defense mechanism of the plant (Sathiyabama et al., 2016), and the expression of the metabolite curcumin as well as key enzymes for curcumin biosynthesis in the tubers was detected based on transcriptomic and metabolomic data. In other words, drought may be one of the reasons for the induction of tuber formation in turmeric.

Taken together, we provide a chromosome-level genome of turmeric, a reference genome of the Zingiberaceae family. The phylogenetic position and a WGD event of the turmeric genome was revealed by comparative genomic analysis. The integration of transcriptomic and metabolomic data improved our understanding of the curcumin biosynthetic pathway and turmeric tuber formation. Moreover, this provides a basis for understanding the relationship between plant survival and secondary metabolite synthesis under drought stress. These data are an important reference for further studies on molecular breeding in turmeric and agricultural production, as well as for comparative genomic analysis of Zingiberaceae family.

## Method

### Sample collection and genome sequencing

*Curcuma longa* was collected in Qianwei County (Leshan, China, coordinates: 104°07′22″E, 029°03′48″N), and total genomic DNA was extracted from the root tissues of *C. longa* following the CTAB method (Raimundo et al., 2018). Based on the Illumina protocols, we constructed two Illumina libraries with fragment size of ~ 350 bp, and the libraries were subjected to paired-end 150 bp (PE 150) sequencing using the Illumina HiSeq X-ten platform (Illumina, San Diego, CA, USA). Then the raw Illumina reads were filtered to obtain clean reads for estimating genome size, GC content, and heterozygosity level. Another library was constructed using the PacBio single-molecule real-time (SMRT) library construction protocol for the second genomic DNA sequencing approach. The genome was sequenced on the PacBio Sequel platform (Pacific Biosciences, Menlo Park, CA, USA). A total of ~224.90 Gb of clean data were used for genome assembly. As a third approach, a Hi-C library was generated and sequenced on a HiSeq X Ten platform (PE 150 bp). ~106.73 Gb Hi-C clean reads were obtained after removing low-quality reads.

### RNA sequencing and gene expression analysis

Rhizomes, tubers and adventitious roots collected from *C. longa* were used for transcriptome analysis, where tubers were collected according to their growth segments at three developmental stages (Tu1-Tu3). Each tissue was collected in three biological replicates. All samples were immediately stored in liquid nitrogen after being rinsed with Milli-Q water. The RNA Prep Pure Plant Kit (TIANGEN, China) was employed to extract total RNA as described by the manufacturer. The Illumina Novaseq 6000 platform was used for RNA sequencing (Illumina, San Diego, CA, USA). Raw reads were trimmed to remove adaptors using Trimmomatic (Bolger et al., 2014). Clean reads were mapped to the genome using HISAT2 (Kim et al., 2015), and then TPM (per million transcripts) was analyzed using StringTie (Pertea et al., 2016). Differentially expressed genes (DEGs) were determined using DEseq2 package (https://bioconductor.org/packages/release/bioc/html/DESeq2.html). The gene expression profiles of different tissues were presented as heat maps using TBtools (Chen et al., 2020).

### Genome survey and genome assembly

We estimated the genome size using k-mer frequencies. Jellyfish (Marçais and Kingsford, 2011) and GenomeScope (Vurture et al., 2017) were used to construct the 21-mer distribution of 98.73 Gb Illumina short reads. As a consequence, we calculated the haploid *C. longa* genome size to be 1.19 Gb, with a heterozygous rate of 3.53%. Canu (Koren et al., 2018) was used for long read error correction, and SMARTdenovo (Liu et al., 2020) was utilized for *de novo* assembly of PacBio reads. Additionally, Purge_Haplotigs pipeline (Roach et al., 2018) was performed to filter the redundant contigs of *de novo* assembly. The Illumina short reads and PacBio long reads were used for further polishing with the NextPolish (Hu et al., 2020) to improve the accuracy of the assembly. The contigs of our draft genome were assembled, ordered, and oriented onto the 21 pseudochromosomes of *C. longa* (2n=3x=63) by ALLHIC (Zhang et al., 2019) using paired-end reads from Hi-C. The completeness of the genome assembly was assessed by Bench-marking Universal Single-Copy Orthologs (BUSCO) v5.0 (Simão et al., 2015) with embryophyta_odb10. We also calculated the LTR assembly Index (LAI) (Ou et al., 2018) using the LTR_Finder and LTR_retriever package to assess genome quality.

### Repeat annotation

*De novo* identification and homology-based searches were used to annotate repeat sequences in the *C. longa* genome. For the *de novo* prediction, a custom repeat library was produced by LTR_Finder (Xu and Wang, 2007) and RepeatModeler (Flynn et al., 2020), and then merged with the known repeat database Repbase (Jurka et al., 2005) to generate the final repeat library. The assembly genome was aligned to the final repeat library using RepeatMasker (Chen, 2004) and for homology−based searches.

### Gene annotation

We combined three different strategies: *ab initio* prediction, homology-based prediction and transcript-based prediction for the protein-coding genes of *C. longa* genome. For *ab initio* predictions, we used Augustus (Stanke and Waack, 2003), and GlimmerHMM (Majoros et al., 2004). Based on homologous comparisons, protein sequences from three related species (*Musa acuminate*, *Musa balbisiana* and *Zingiber officinale*) were provided as protein evidence using Exonerate v2.2.0 (Slater and Birney, 2005). Then, the RNA-sequencing data from five tissue of *C. longa* were assembled into transcripts by Trinity (Grabherr et al., 2011) to perform transcript-based prediction and the gene structure was further predicted using PASA (Haas et al., 2003). Finally, EVidenceModeler (Haas et al., 2008) was employed to integrate the prediction results of three strategies. The functional annotation of protein-coding genes was by BLASTP (Camacho et al., 2009) (e-value cutoff of 1e-5) against non-redundant protein sequence (NR) (Aron et al., 2011), Kyoto Encyclopedia of Genes and Genomes (KEGG) (Ogata et al., 1999), Swiss-Prot (Boeckmann et al., 2003), KOG and TrEMBL (Boeckmann et al., 2003) databases.

### Noncoding RNA prediction

For noncoding RNA annotations, the whole *C. longa* genome was scanned for microRNAs and rRNAs using BLASTN based on the Rfam (Kalvari et al., 2018) database. tRNAs were predicted by tRNAscan-SE (Lowe and Eddy, 1997).

### Comparative genomics analyses

The protein-coding genes from *C. longa* and eight other species (*Arabidopsis thaliana*, *Oryza sativa*, *Phoenix dactylifera*, *Zea mays*, *Ensete glaucum*, *Musa acuminate*, *Musa balbisiana*, *Zingiber officinale*) were clustered into orthologous groups using OrthoFinder v2.3.7 (Emms and Kelly, 2019). The longest protein sequences were employed to perform all-against-all comparisons using Diamond (E-value ≤ 1e−5). The single-copy protein sequences were aligned by MAFFT (Katoh et al., 2002), and positions showing poor alignment were eliminated with Gblocks v0.91b (Castresana, 2000). IQ-TREE v2.0.7 (Nguyen et al., 2015) constructed the phylogenetic trees with JTT+G+F model. The bootstrap support values were calculated on 1000 replicates. MCMCTREE (Yang, 2007) was applied to calculate divergence time of above species with parameters (burnin=5,000,000, nsample=5,000,000 and sampfreq=30). Two calibration points from the TimeTree database (http://www.timetree.org/) were selected as normal priors to constrain the age of the nodes, such as published divergence times for *M. acuminate* - *E. glaucum* (~ 56–69 Mya), *O. sativa* - *Z. mays* (~ 42–52 Mya). CAFE5 (Mendes et al., 2021) was used to calculate the expansion and contraction of OrthoFinder-derived gene clusters based on changes in gene family size. Functional enrichment analysis of these expanded and contracted genes in *C. longa* genome was performed using the clusterProfiler R package (Yu et al., 2012b).

### Genome synteny and synonymous substitution rates (Ks) analysis

All-against-all comparisons BLASTP (E-value ≤ 1e−5) analyses of proteins were conducted between and within the three species (*C. longa*, *Z. officinale* and *M. acuminate*). The distribution of the synonymous substitution rate (Ks) among orthologs (between *C. longa-Z. officinale*, *C. longa-M. acuminate* and *Z. officinale -M. acuminate*) and paralogs (within the *C. longa*, *Z. officinale* and *M. acuminate* genome) revealed species differentiation and whole-genome duplication events. MCScanX (Wang et al., 2012) was used to identify syntenic blocks using BLASTP results and genome annotation files for each species. The Ks values of ortholog pairs or paralog pairs were plotted using the WGDI (https://pypi.org/project/wgdi/), where the Ks distribution was used to evaluate the WGD events, and the divergence time was calculated using the formula T = Ks/2r, where r is the neutral substitution rate.

### Identification and analysis of polyketide synthases (PKS) genes

We selected three species (*Z. officinale*, *M. acuminate* and *O. sativa*) for comparative analysis of the PKS gene family together with turmeric. Using HMMER (Eddy, 2009) with the Pfam protein family database (http://pfam.xfam.org/), and genes containing the Chal_sti_synt_N (PF00195) domain and Chal_sti_synt_C (PF02797) domain (E-value ≤ 1e−5) were considered as candidate PKSs. The putative protein sequences of all PKS genes were aligned using MAFFT and then a phylogenetic tree was constructed using IQTREE with 1000 bootstrap replicates.

### UPLC-MS/MS analysis of curcumin

Turmeric rhizomes and tubers were collected at maturity and detected for curcumin using a Waters ACQUITY UPLC coupled with a XEVO QTOF mass spectrometer (Waters Corporation, Milford, MA, USA). The analytical conditions were as follows: solvent system of acetonitrile(A), water (0.1% formic acid, B); 0–10min, 5%–15% A, then 10–20 min, 15%–30% B, and 20–30 min, 30%–70% B; flow rate, 0.2 mL/min; temperature, 35°C; injection volume, 1 μL; scanning mode, positive and negative ion mode.

## Data availability statement

The data presented in the study are deposited in the China National GeneBank DataBase (CNGBdb) https://db.cngb.org/, accession number CNP0003456.

## Author contributions

CP and JG designed the project. LZ, XZ, and QL collected plant materials. YY, XS, and XY conducted experiments. YY and SG analyzed data and conducted bioinformatic analysis. YY, XX, and JG wrote and modified the manuscript. All authors contributed to the article and approved the submitted version.

## Funding

This work was supported by National Interdisciplinary Innovation Team of Traditional Chinese Medicine (ZYYCXTD-D-202209), Major increase and decrease of expenditure at the central level (2060302), Talent Project in Chengdu University of TCM (QNXZ2018017, QNXZ2019001), Multi-dimensional evaluation of characteristic traditional Chinese medicine resources and product development innovation team (2022C001), and Science and Technology Planning Project of Sichuan Provincial Department of Science and Technology (2020YFN0152, 22CXTD0009).

## Conflict of interest

The authors declare that the research was conducted in the absence of any commercial or financial relationships that could be construed as a potential conflict of interest.

## Publisher’s note

All claims expressed in this article are solely those of the authors and do not necessarily represent those of their affiliated organizations, or those of the publisher, the editors and the reviewers. Any product that may be evaluated in this article, or claim that may be made by its manufacturer, is not guaranteed or endorsed by the publisher.

## References

[B1] Abelenda J. A. Prat S. (2013). Cytokinins: Determinants of sink storage ability. Curr. biol.: CB 23, R561–R563. doi: 10.1016/j.cub.2013.05.020 23845242

[B2] Ageez A. Kazama Y. Sugiyama R. Kawano S. (2005). Male-Fertility genes expressed in male flower buds of silene latifolia include homologs of anther-specific genes. Genes Genet. Syst. 80, 403–413. doi: 10.1266/ggs.80.403 16501309

[B3] Aksenova N. Konstantinova T. Golyanovskaya S. Sergeeva L. Romanov G. (2012). Hormonal regulation of tuber formation in potato plants. Russian J. Plant Physiol. 59, 451–466. doi: 10.1134/S1021443712040024

[B4] Anamthawat-Jónsson K. Umpunjun P. (2020). Polyploidy in the ginger family from Thailand. Chromosomal Abnormalities 8, 115–129. doi: 10.5772/intechopen.92859

[B5] Aron M. B. Lu S. Anderson J. B. Farideh C. Derbyshire M. K. Carol D. W. S.. (2011). CDD: A conserved domain database for the functional annotation of proteins. Nucleic Acids Res. 39, D225–D229. doi: 10.1093/nar/gkq1189. 21109532 PMC3013737

[B6] Austin M. B. Noel J. P. (2003). The chalcone synthase superfamily of type III polyketide synthases. Natural prod. Rep. 20 (1), 79–110. doi: 10.1039/b100917f 12636085

[B7] Blanc G. Wolfe K. H. (2004). Widespread paleopolyploidy in model plant species inferred from age distributions of duplicate genes. Plant Cell 16, 1667–1678. doi: 10.1105/tpc.021345 15208399 PMC514152

[B8] Boeckmann B. Bairoch A. Apweiler R. Blatter M.-C. Estreicher A. Gasteiger E.. (2003). The SWISS-PROT protein knowledgebase and its supplement TrEMBL in 2003. Nucleic Acids Res. 31, 365–370. doi: 10.1093/nar/gkg095 12520024 PMC165542

[B9] Bolger A. M. Lohse M. Usadel B. (2014). Trimmomatic: A flexible trimmer for illumina sequence data. Bioinformatics 30, 2114–2120. doi: 10.1093/bioinformatics/btu170 24695404 PMC4103590

[B10] Breia R. Conde A. Badim H. Fortes A. M. Gerós H. Granell A. (2021). Plant SWEETs: From sugar transport to plant–pathogen interaction and more unexpected physiological roles. Plant Physiol. 186, 836–852. doi: 10.1093/plphys/kiab127 33724398 PMC8195505

[B11] Camacho C. G. Coulouris G. Avagyan V. Ma N. Papadopoulos J. (2009). BLAST plus: Architecture and applications. BMC Bioinf. 10, 1–9. doi: 10.1186/1471-2105-10-421 PMC2803857 20003500

[B12] Castresana J. (2000). Selection of conserved blocks from multiple alignments for their use in phylogenetic analysis. Mol. Biol. Evol. 17, 540–552. doi: 10.1093/oxfordjournals.molbev.a026334 10742046

[B13] Chakraborty A. Mahajan S. Jaiswal S. K. Sharma V. K. (2021). Genome sequencing of turmeric provides evolutionary insights into its medicinal properties. Commun. Biol. 4, 1–12. doi: 10.1038/s42003-021-02720-y 34654884 PMC8521574

[B14] Chang J. Li X. Fu W. Wang J. Yong Y. Shi H.. (2019). Asymmetric distribution of cytokinins determines root hydrotropism in arabidopsis thaliana. Cell Res. 29, 984–993. doi: 10.1038/s41422-019-0239-3 31601978 PMC6951336

[B15] Chen N. (2004). Using RepeatMasker to identify repetitive elements in genomic sequences. Curr. Protoc. Bioinf 5 (1), 4–10. doi: 10.1002/0471250953.bi0410s05 18428725

[B16] Chen L. Q. (2014). SWEET sugar transporters for phloem transport and pathogen nutrition. New Phytol. 201, 1150–1155. doi: 10.1111/nph.12445 24649486

[B17] Chen C. Chen H. Zhang Y. Thomas H. R. Frank M. H. He Y.. (2020). TBtools: An integrative toolkit developed for interactive analyses of big biological data. Mol. Plant 13, 1194–1202. doi: 10.1016/j.molp.2020.06.009 32585190

[B18] Cheng S.-P. Jia K.-H. Liu H. Zhang R.-G. Li Z.-C. Zhou S.-S.. (2021). Haplotype-resolved genome assembly and allele-specific gene expression in cultivated ginger. Hortic. Res. 8, 188. doi: 10.1038/s41438-021-00599-8 34354050 PMC8342452

[B19] Chen J. Xia N. Zhao J. Chen J. Henny R. J. (2013). Chromosome numbers and ploidy levels of Chinese curcuma species. Hortscience 48, 525–530. doi: 10.21273/HORTSCI.48.5.525

[B20] Chen X. Yao Q. Gao X. Jiang C. Harberd N. P. Fu X. (2016). Shoot-to-root mobile transcription factor HY5 coordinates plant carbon and nitrogen acquisition. Curr. Biol. 26, 640–646. doi: 10.1016/j.cub.2015.12.066 26877080

[B21] Chintakovid N. Tisarum R. Samphumphuang T. Sotesaritkul T. Cha-Um S. (2022). Evaluation of curcuminoids, physiological adaptation, and growth of curcuma longa under water deficit and controlled temperature. Protoplasma 259, 301–315. doi: 10.1007/s00709-021-01670-w 34023960

[B22] Collin A. Daszkowska-Golec A. Szarejko I. (2021). Updates on the role of abscisic acid insensitive 5 (abi5) and abscisic acid-responsive element binding factors (abfs) in ABA signaling in different developmental stages in plants. Cells 10 (8), 1996. doi: 10.3390/cells10081996 34440762 PMC8394461

[B23] Dao T. T. H. Linthorst H. J. M. Verpoorte R. (2011). Chalcone synthase and its functions in plant resistance. Phytochem. Rev. 10, 397–412. doi: 10.1007/s11101-011-9211-7 21909286 PMC3148432

[B24] D’hont A. Denoeud F. Aury J.-M. Baurens F.-C. Carreel F. Garsmeur O.. (2012). The banana (Musa acuminata) genome and the evolution of monocotyledonous plants. Nature 488, 213–217. doi: 10.1038/nature11241 22801500

[B25] Ding Z. Fu L. Tie W. Yan Y. Wu C. Dai J.. (2020). Highly dynamic, coordinated, and stage-specific profiles are revealed by a multi-omics integrative analysis during tuberous root development in cassava. J. Exp. Bot. 71, 7003–7017. doi: 10.1093/jxb/eraa369 32777039

[B26] Dosoky N. S. Satyal P. Setzer W. N. (2019). Variations in the volatile compositions of species. Foods 8 (2), 53. doi: 10.3390/foods8020053 PMC6406329 30717336

[B27] Dosoky N. S. Setzer W. N. (2018). Chemical composition and biological activities of essential oils of species. Nutrients 10 (9), 1196. doi: 10.3390/nu10091196 PMC6164907 30200410

[B28] Eddy S. R. (2009). A new generation of homology search tools based on probabilistic inference,” in Genome inform 23, 205–211. doi: 10.1142/9781848165632_0019 20180275

[B29] Ehrlich J. J. Weerts R. M. Sayane S. Culbertson A. T. Honzatko R. B. Jernigan R. L.. Xyloglucan xylosyltransferase 1 displays promiscuity toward donor substrates during *In vitro* reactions. Plant Cell Physiol 62 (12), 1890–1901. doi: 10.1093/pcp/pcab114. 34265062

[B30] Emms D. M. Kelly S. (2019). OrthoFinder: Phylogenetic orthology inference for comparative genomics. Genome Biol. 20, 238. doi: 10.1186/s13059-019-1832-y 31727128 PMC6857279

[B31] Eveland A. L. Jackson D. P. (2012). Sugars, signalling, and plant development. J. Exp. Bot. 63, 3367–3377. doi: 10.1093/jxb/err379 22140246

[B32] Fabianowska-Majewska K. Kaufman-Szymczyk A. Szymanska-Kolba A. Jakubik J. Majewski G. Lubecka K. (2021). Curcumin from turmeric rhizome: A potential modulator of DNA methylation machinery in breast cancer inhibition. Nutrients 13 (2), 332. doi: 10.3390/nu13020332 33498667 PMC7910847

[B33] Flynn J. M. Hubley R. Goubert C. Rosen J. Clark A. G. Feschotte C.. (2020). RepeatModeler2 for automated genomic discovery of transposable element families. Proc. Natl. Acad. Sci. United States America 117, 9451–9457. doi: 10.1073/pnas.1921046117 PMC7196820 32300014

[B34] Fulton D. C. Edwards A. Pilling E. Robinson H. L. Fahy B. Seale R.. (2002). Role of granule-bound starch synthase in determination of amylopectin structure and starch granule morphology in potato. J. Biol. Chem. 277, 10834–10841. doi: 10.1074/jbc.M111579200 11801600

[B35] Geng D. Shen X. Xie Y. Yang Y. Bian R. Gao Y.. (2020). Regulation of phenylpropanoid biosynthesis by MdMYB88 and MdMYB124 contributes to pathogen and drought resistance in apple. Hortic. Res. 7, 102. doi: 10.1038/s41438-020-0324-2 32637130 PMC7327078

[B36] Gibson S. I. (2005). Control of plant development and gene expression by sugar signaling. Curr. Opin. Plant Biol. 8, 93–102. doi: 10.1016/j.pbi.2004.11.003 15653406

[B37] Grabherr M. G. Haas B. J. Yassour M. Levin J. Z. Thompson D. A. Amit I.. (2011). Full-length transcriptome assembly from RNA-seq data without a reference genome. Nat. Biotechnol. 29, 644–652. doi: 10.1038/nbt.1883 21572440 PMC3571712

[B38] Gullner G. Komives T. Király L. Schröder P. (2018). Glutathione s-transferase enzymes in plant-pathogen interactions. Front. Plant Sci. 9, 1836. doi: 10.3389/fpls.2018.01836 30622544 PMC6308375

[B39] Gupta S. C. Sung B. Kim J. H. Prasad S. Li S. Aggarwal B. B. (2013). Multitargeting by turmeric, the golden spice: From kitchen to clinic. Mol. Nutr. Food Res. 57, 1510–1528. doi: 10.1002/mnfr.201100741 22887802

[B40] Gu H. Wang Y. Xie H. Qiu C. Zhang S. Xiao J.. (2020). Drought stress triggers proteomic changes involving lignin, flavonoids and fatty acids in tea plants. Sci. Rep. 10, 1–11. doi: 10.1038/s41598-020-72596-1 32968186 PMC7511325

[B41] Haas B. J. Delcher A. L. Mount S. M. Wortman J. R. Smith R. K. Hannick L. I.. (2003). Improving the arabidopsis genome annotation using maximal transcript alignment assemblies. Nucleic Acids Res. 31, 5654–5666. doi: 10.1093/nar/gkg770 14500829 PMC206470

[B42] Haas B. J. Salzberg S. L. Zhu W. Pertea M. Allen J. E. Orvis J.. (2008). Automated eukaryotic gene structure annotation using EVidenceModeler and the program to assemble spliced alignments. Genome Biol. 9, R7. doi: 10.1186/gb-2008-9-1-r7 18190707 PMC2395244

[B43] Hartmann A. Senning M. Hedden P. Sonnewald U. Sonnewald S. (2011). Reactivation of meristem activity and sprout growth in potato tubers require both cytokinin and gibberellin. Plant Physiol. 155, 776–796. doi: 10.1104/pp.110.168252 21163959 PMC3032466

[B44] Hu J. Fan J. Sun Z. Liu S. (2020). NextPolish: A fast and efficient genome polishing tool for long-read assembly. Bioinf. (Oxford England) 36, 2253–2255. doi: 10.1093/bioinformatics/btz891 31778144

[B45] Jiang C. Kim S. Y. Suh D.-Y. (2008). Divergent evolution of the thiolase superfamily and chalcone synthase family. Mol. Phylogenet. Evol. 49, 691–701. doi: 10.1016/j.ympev.2008.09.002 18824113

[B46] Jogawat A. Yadav B. Chhaya Lakra N. Singh A. K. Narayan O. P. (2021). Crosstalk between phytohormones and secondary metabolites in the drought stress tolerance of crop plants: A review. Physiol plant. 172, 1106–1132. doi: 10.1111/ppl.13328 33421146

[B47] Jurka J. Kapitonov V. V. Pavlicek A. Klonowski P. Kohany O. Walichiewicz J. (2005). Repbase update, a database of eukaryotic repetitive elements. Cytogenet. Genome Res. 110, 462–467. doi: 10.1159/000084979 16093699

[B48] Kalvari I. Nawrocki E. P. Argasinska J. Quinones-Olvera N. Finn R. D. Bateman A.. (2018). Non-coding RNA analysis using the rfam database. Curr. Protoc. Bioinf. 62, e51. doi: 10.1002/cpbi.51 PMC6754622 29927072

[B49] Katoh K. Misawa K. Kuma K. I. Miyata T. (2002). MAFFT: A novel method for rapid multiple sequence alignment based on fast Fourier transform. Nucleic Acids Res. 30, 3059–3066. doi: 10.1093/nar/gkf436 12136088 PMC135756

[B50] Katsuyama Y. Kita T. Funa N. Horinouchi S. (2009). Curcuminoid biosynthesis by two type III polyketide synthases in the herb curcuma longa. J. Biol. Chem. 284, 11160–11170. doi: 10.1074/jbc.M900070200 19258320 PMC2670121

[B51] Kim D. Langmead B. Salzberg S. L. (2015). HISAT: A fast spliced aligner with low memory requirements. Nat. Methods 12, 357–360. doi: 10.1038/nmeth.3317 25751142 PMC4655817

[B52] Kocaadam B. Şanlier N. (2017). Curcumin, an active component of turmeric (Curcuma longa), and its effects on health. Crit. Rev. Food Sci. Nutr. 57, 2889–2895. doi: 10.1080/10408398.2015.1077195 26528921

[B53] Kondhare K. R. Natarajan B. Banerjee A. K. (2020). Molecular signals that govern tuber development in potato. Int. J. Dev. Biol. 64, 133–140. doi: 10.1387/ijdb.190132ab 32659001

[B54] Kondhare K. R. Patil A. B. Giri A. P. (2021). Auxin: An emerging regulator of tuber and storage root development. Plant Sci. 306, 110854. doi: 10.1016/j.plantsci.2021.110854 33775360

[B55] Kong W. An B. Zhang Y. Yang J. Li S. Sun T.. (2019). Sugar transporter proteins (STPs) in gramineae crops: Comparative analysis, phylogeny, evolution, and expression profiling. Cells 8, 560. doi: 10.3390/cells8060560 PMC6628381 31181814

[B56] Koren S. Rhie A. Walenz B. P. Dilthey A. T. Bickhart D. M. Kingan S. B.. (2018). *De novo* assembly of haplotype-resolved genomes with trio binning. Nat. Biotechnol. 36, 1174–1182. doi: 10.1038/nbt.4277 PMC6476705 30346939

[B57] Kress W. J. Prince L. M. Williams K. J. (2002). The phylogeny and a new classification of the gingers (Zingiberaceae): Evidence from molecular data. Am. J. Bot. 89, 1682–1696. doi: 10.3732/ajb.89.10.1682 21665595

[B58] Kroymann J. (2011). Natural diversity and adaptation in plant secondary metabolism. Curr. Opin. Plant Biol. 14, 246–251. doi: 10.1016/j.pbi.2011.03.021 21514879

[B59] Kulik A. Wawer I. Krzywińska E. Bucholc M. Dobrowolska G. (2011). SnRK2 protein kinases–key regulators of plant response to abiotic stresses. Omics: J. Integr. Biol. 15, 859–872. doi: 10.1089/omi.2011.0091 PMC3241737 22136638

[B60] Kurowska M. M. (2020). TIP aquaporins in plants: Role in abiotic stress tolerance. Abiotic Stress Plants 20, 423–443. doi: 10.5772/intechopen.94165

[B61] Lee Y. Nishizawa T. Takemoto M. Kumazaki K. Yamashita K. Hirata K.. (2017). Structure of the triose-phosphate/phosphate translocator reveals the basis of substrate specificity. Nat. Plants 3, 825–832. doi: 10.1038/s41477-017-0022-8 28970497

[B62] Leong-Skornicková J. Sída O. Jarolímová V. Sabu M. Fér T. Trávnícek P.. (2007). Chromosome numbers and genome size variation in Indian species of curcuma (Zingiberaceae). Ann. Bot. 100, 505–526. doi: 10.1093/aob/mcm144 17686760 PMC2533610

[B63] Liang H. Zhang Y. Deng J. Gao G. Ding C. Zhang L.. (2020). The complete chloroplast genome sequences of 14 species: Insights into genome evolution and phylogenetic relationships within zingiberales. Front. Genet. 11, 802. doi: 10.3389/fgene.2020.00802 32849804 PMC7396571

[B64] Liao W. Zhang Q. Zhang L. Dong F. Liu F. He Y.. (2020). Qualitative and quantitative analysis of trace element of terpenoid conjugated curcuminoid and curcuminoids in herbal medicine derived from different curcuma species. Chin. Trad. Herbal Drugs 51, 1076–1081. doi: 10.7501/j.issn.0253-2670.2020.04.035

[B65] Licausi F. Ohme-Takagi M. Perata P. (2013). APETALA 2/Ethylene responsive factor (AP 2/ERF) transcription factors: Mediators of stress responses and developmental programs. New Phytol. 199, 639–649. doi: 10.1111/nph.12291 24010138

[B66] Li J. Dai X. Zhao Y. (2006). A role for auxin response factor 19 in auxin and ethylene signaling in arabidopsis. Plant Physiol. 140, 899–908. doi: 10.1104/pp.105.070987 16461383 PMC1400570

[B67] Liebthal M. Maynard D. Dietz K.-J. (2018). Peroxiredoxins and redox signaling in plants. Antioxid Redox Signaling 28, 609–624. doi: 10.1089/ars.2017.7164 PMC5806080 28594234

[B68] Li D. Ono N. Sato T. Sugiura T. Altaf-Ul-Amin M. Ohta D.. (2015). Targeted integration of RNA-seq and metabolite data to elucidate curcuminoid biosynthesis in four curcuma species. Plant Cell Physiol. 56, 843–851. doi: 10.1093/pcp/pcv008 25637373

[B69] Liu H. Wu S. Li A. Ruan J. (2020). SMARTdenovo: A *de novo* assembler using long noisy reads. Preprints 2020, 2020090207doi: 10.20944/preprints202009.0207.v1 PMC9632051 36824332

[B70] Li H.-L. Wu L. Dong Z. Jiang Y. Jiang S. Xing H.. (2021b). Haplotype-resolved genome of diploid ginger (Zingiber officinale) and its unique gingerol biosynthetic pathway. Hortic. Res. 8, 189. doi: 10.1038/s41438-021-00627-7 34354044 PMC8342499

[B71] Lowe T. M. Eddy S. R. (1997). tRNAscan-SE: A program for improved detection of transfer RNA genes in genomic sequence. Nucleic Acids Res. 25, 955–964. doi: 10.1093/nar/25.5.955 9023104 PMC146525

[B72] Luthje S. Doring O. Heuer S. Luthen H. Bottger M. (1997). Oxidoreductases in plant plasma membranes. Biochim. Biophys. Acta-Biomembranes-Including Rev. Biomembr. 1331, 81–102. doi: 10.1016/S0304-4157(96)00016-0 9325436

[B73] Lv X. Zhang Y. Zhang Y. Fan S. Kong L. (2020). Source-sink modifications affect leaf senescence and grain mass in wheat as revealed by proteomic analysis. BMC Plant Biol. 20, 1–17. doi: 10.1186/s12870-020-02447-8 32503423 PMC7275590

[B74] Majoros W. H. Pertea M. Salzberg S. L. (2004). TigrScan and GlimmerHMM: Two open source ab initio eukaryotic gene-finders. Bioinf. (Oxford England) 20, 2878–2879. doi: 10.1093/bioinformatics/bth315 15145805

[B75] Marçais G. Kingsford C. (2011). A fast, lock-free approach for efficient parallel counting of occurrences of k-mers. Bioinformatics 27, 764–770. doi: 10.1093/bioinformatics/btr011 21217122 PMC3051319

[B76] Mendes F. K. Vanderpool D. Fulton B. Hahn M. W. (2021). CAFE 5 models variation in evolutionary rates among gene families. Bioinformatics 36, 5516–5518. doi: 10.1093/bioinformatics/btaa1022 33325502

[B77] Nguyen L.-T. Schmidt H. A. Von Haeseler A. Minh B. Q. (2015). IQ-TREE: A fast and effective stochastic algorithm for estimating maximum-likelihood phylogenies. Mol. Biol. Evol. 32, 268–274. doi: 10.1093/molbev/msu300 25371430 PMC4271533

[B78] Obata T. Fernie A. R. (2012). The use of metabolomics to dissect plant responses to abiotic stresses. Cell. Mol. Life sci.: CMLS 69, 3225–3243. doi: 10.1007/s00018-012-1091-5 22885821 PMC3437017

[B79] Ogata H. Goto S. Sato K. Fujibuchi W. Bono H. Kanehisa M. (1999). KEGG: Kyoto encyclopedia of genes and genomes. Nucleic Acids Res. 27, 29–34. doi: 10.1093/nar/27.1.29 9847135 PMC148090

[B80] Ou S. Chen J. Jiang N. (2018). Assessing genome assembly quality using the LTR assembly index (LAI). Nucleic Acids Res. 46, e126–e126. doi: 10.1093/nar/gky730 30107434 PMC6265445

[B81] Pertea M. Kim D. Pertea G. M. Leek J. T. Salzberg S. L. (2016). Transcript-level expression analysis of RNA-seq experiments with HISAT, StringTie and ballgown. Nat. Protoc. 11, 1650–1667. doi: 10.1038/nprot.2016.095 27560171 PMC5032908

[B82] Pothiraj R. Ravikumar M. J. Suthanthiram B. Subbaraya U. Krishnamurthy P. (2021). Genome-scale analyses of polyketide synthases in banana: Phylogenetics and expression profiling forecast their candidacy in specialized metabolism. Gene 778, 145472. doi: 10.1016/j.gene.2021.145472 33549715

[B83] Prasad S. Gupta S. C. Tyagi A. K. Aggarwal B. B. (2014). Curcumin, a component of golden spice: From bedside to bench and back. Biotechnol. Adv. 32, 1053–1064. doi: 10.1016/j.biotechadv.2014.04.004 24793420

[B84] Raimundo J. Reis C. M. G. Ribeiro M. M. (2018). Rapid, simple and potentially universal method for DNA extraction from opuntia spp. fresh cladode tissues suitable for PCR amplification. Mol. Biol. Rep. 45, 1405–1412. doi: 10.1007/s11033-018-4303-8 30109548

[B85] Ramirez-Ahumada M. D. C. Timmermann B. N. Gang D. R. (2006). Biosynthesis of curcuminoids and gingerols in turmeric (Curcuma longa) and ginger (Zingiber officinale): Identification of curcuminoid synthase and hydroxycinnamoyl-CoA thioesterases. Phytochemistry 67, 2017–2029. doi: 10.1016/j.phytochem.2006.06.028 16890967

[B86] Ravi V. Naskar S. K. Makeshkumar T. Babu B. Prakash Krishnan B. S. (2009). Molecular physiology of storage root formation and development in sweet potato (Ipomoea batatas (L.) lam.). J Root Crops 35 (1), 1–27.

[B87] Rezvanirad A. Mardani M. Shirzad H. Ahmadzadeh S. M. Mahmoudi G. (2016). Curcuma longa: A review of therapeutic effects in traditional and modern medical references. J Chem Pharmaceut Sci 9 (4), 3438–3448.

[B88] Riesmeier J. W. Willmitzer L. Frommer W. B. (1994). Evidence for an essential role of the sucrose transporter in phloem loading and assimilate partitioning. EMBO J. 13, 1–7. doi: 10.1002/j.1460-2075.1994.tb06229.x 8306952 PMC394773

[B89] Roach M. J. Schmidt S. A. Borneman A. R. (2018). Purge haplotigs: Allelic contig reassignment for third-gen diploid genome assemblies. BMC Bioinf. 19, 1–10. doi: 10.1186/s12859-018-2485-7 PMC6267036 30497373

[B90] Roumeliotis E. Kloosterman B. Oortwijn M. Kohlen W. Bouwmeester H. J. Visser R. G.. (2012). The effects of auxin and strigolactones on tuber initiation and stolon architecture in potato. J. Exp. Bot. 63, 4539–4547. doi: 10.1093/jxb/ers132 22689826 PMC3421988

[B91] Santiago J. Dupeux F. Round A. Antoni R. Park S.-Y. Jamin M.. (2009). The abscisic acid receptor PYR1 in complex with abscisic acid. Nature 462, 665–668. doi: 10.1038/nature08591 19898494

[B92] Sathiyabama M. Bernstein N. Anusuya S. (2016). Chitosan elicitation for increased curcumin production and stimulation of defence response in turmeric (Curcuma longa l.). Ind. Crops Prod. 89, 87–94. doi: 10.1016/j.indcrop.2016.05.007

[B93] Sharma R. A. Gescher A. J. Steward W. P. (2005). Curcumin: The story so far. Eur. J. Cancer (Oxford England: 1990) 41, 1955–1968. doi: 10.1016/j.ejca.2005.05.009 16081279

[B94] Shehzad A. Qureshi M. Anwar M. N. Lee Y. S. (2017). Multifunctional curcumin mediate multitherapeutic effects. J. Food Sci. 82, 2006–2015. doi: 10.1111/1750-3841.13793 28771714

[B95] Simão F. A. Waterhouse R. M. Ioannidis P. Kriventseva E. V. Zdobnov E. M. (2015). BUSCO: Assessing genome assembly and annotation completeness with single-copy orthologs. Bioinf. (Oxford England) 31, 3210–3212. doi: 10.1093/bioinformatics/btv351 26059717

[B96] Slater G. S. C. Birney E. (2005). Automated generation of heuristics for biological sequence comparison. BMC Bioinf. 6, 31. doi: 10.1186/1471-2105-6-31 PMC553969 15713233

[B97] Stanke M. Waack S. (2003). Gene prediction with a hidden Markov model and a new intron submodel. Bioinf. (Oxford England) 19 Suppl 2, ii215–ii225. doi: 10.1093/bioinformatics/btg1080 14534192

[B98] Stebbins G. L. (1966). Chromosomal variation and evolution: Polyploidy and chromosome size and number shed light on evolutionary processes in higher plants. Science 152, 1463–1469. doi: 10.1126/science.152.3728.1463 17788022

[B99] Sun J. Bu J. Zhao H. Mao Y. Zeng W. Guo J.. (2018). Multivariate data analysis of volatile metabolites in rhizomes and radixes of four medicinal plants from curcuma l. Acta Pharm. Sin. 53, 1215–1224. doi: 10.16438/j.0513-4870.2018-0408

[B100] Sun W. Wang S. Zhao W. Wu C. Guo S. Gao H.. (2017). Chemical constituents and biological research on plants in the genus curcuma. Crit. Rev. Food Sci. Nutr. 57, 1451–1523. doi: 10.1080/10408398.2016.1176554 27229295

[B101] Van De Peer Y. Fawcett J. A. Proost S. Sterck L. Vandepoele K. (2009a). The flowering world: A tale of duplications. Trends Plant Sci. 14, 680–688. doi: 10.1016/j.tplants.2009.09.001 19818673

[B102] Van De Peer Y. Maere S. Meyer A. (2009b). The evolutionary significance of ancient genome duplications. Nat. Rev. Genet. 10, 725–732. doi: 10.1038/nrg2600 19652647

[B103] Van Moerkercke A. Duncan O. Zander M. Šimura J. Broda M. Vanden Bossche R.. (2019). A MYC2/MYC3/MYC4-dependent transcription factor network regulates water spray-responsive gene expression and jasmonate levels. Proc. Natl. Acad. Sci. 116, 23345–23356. doi: 10.1073/pnas.1911758116 31662474 PMC6859355

[B104] Vurture G. W. Sedlazeck F. J. Nattestad M. Underwood C. J. Fang H. Gurtowski J.. (2017). GenomeScope: Fast reference-free genome profiling from short reads. Bioinformatics 33, 2202–2204. doi: 10.1093/bioinformatics/btx153 28369201 PMC5870704

[B105] Wang Y. Tang H. Debarry J. D. Tan X. Li J. Wang X.. (2012). MCScanX: A toolkit for detection and evolutionary analysis of gene synteny and collinearity. Nucleic Acids Res. 40, e49–e49. doi: 10.1093/nar/gkr1293 22217600 PMC3326336

[B106] Woodward A. W. Bartel B. (2005). Auxin: Regulation, action, and interaction. Ann. Bot. 95, 707–735. doi: 10.1093/aob/mci083 15749753 PMC4246732

[B107] Wu P. Guo J. Wang X. Li Q. Zeng J. Hua H.. (2019). Effects of environment factors on yield and constituents related to quality of curcuma longa. J. Chin. Med. Mater. 42, 1969–1972. doi: 10.13863/j.issn1001-4454.2019.09.001

[B108] Wu W. Yang Y.-L. He W.-M. Rouard M. Li W.-M. Xu M.. (2016). Whole genome sequencing of a banana wild relative musa itinerans provides insights into lineage-specific diversification of the musa genus. Sci. Rep. 6, 31586. doi: 10.1038/srep31586 27531320 PMC4987669

[B109] Xu Z. Wang H. (2007). LTR_FINDER: An efficient tool for the prediction of full-length LTR retrotransposons. Nucleic Acids Res. 35, W265–W268. doi: 10.1093/nar/gkm286 17485477 PMC1933203

[B110] Yang Z. (2007). PAML 4: Phylogenetic analysis by maximum likelihood. Mol. Biol. Evol. 24, 1586–1591. doi: 10.1093/molbev/msm088 17483113

[B111] Yan J. Zhang C. Gu M. Bai Z. Zhang W. Qi T.. (2009). The arabidopsis CORONATINE INSENSITIVE1 protein is a jasmonate receptor. Plant Cell 21, 2220–2236. doi: 10.1105/tpc.109.065730 19717617 PMC2751961

[B112] Yoon J. Cho L.-H. Tun W. Jeon J.-S. An G. (2021). Sucrose signaling in higher plants. Plant Sci. 302, 110703. doi: 10.1016/j.plantsci.2020.110703 33288016

[B113] Yoshida H. Tanimoto E. Hirai T. Miyanoiri Y. Mitani R. Kawamura M.. (2018). Evolution and diversification of the plant gibberellin receptor GID1. Proc. Natl. Acad. Sci. 115, E7844–E7853. doi: 10.1073/pnas.1806040115 30068603 PMC6099883

[B114] Yu G. Wang L.-G. Han Y. He Q.-Y. (2012b). clusterProfiler: An R package for comparing biological themes among gene clusters. Omics: J. Integr. Biol. 16, 284–287. doi: 10.1089/omi.2011.0118 PMC3339379 22455463

[B115] Yu D. Xu F. Zeng J. Zhan J. (2012a). Type III polyketide synthases in natural product biosynthesis. IUBMB Life 64, 285–295. doi: 10.1002/iub.1005 22362498

[B116] Záveská E. Fér T. Šída O. Marhold K. Leong-Škorničková J. (2016). Hybridization among distantly related species: Examples from the polyploid genus curcuma (Zingiberaceae). Mol. Phylogenet. Evol. 100, 303–321. doi: 10.1016/j.ympev.2016.04.017 27090448

[B117] Zhang M. Liu R. Liu H. Yang H. Li X. Wang P.. (2021). Citrus NIP5; 1 aquaporin regulates cell membrane water permeability and alters PIPs plasma membrane localization. Plant Mol. Biol. 106, 449–462. doi: 10.1007/s11103-021-01164-6 34173150

[B118] Zhang L. Wei J. Yang Z. Chen F. Xian Q. Su P.. (2018). Distribution and diversity of twelve curcuma species in China. Natural prod. Res. 32, 327–330. doi: 10.1080/14786419.2017.1350667 28722473

[B119] Zhang X. Zhang S. Zhao Q. Ming R. Tang H. (2019). Assembly of allele-aware, chromosomal-scale autopolyploid genomes based on Hi-c data. Nat. Plants 5, 833–845. doi: 10.1038/s41477-019-0487-8 31383970

[B120] Zhu J.-K. (2016). Abiotic stress signaling and responses in plants. Cell 167, 313–324. doi: 10.1016/j.cell.2016.08.029 27716505 PMC5104190

[B121] Zierer W. Rüscher D. Sonnewald U. Sonnewald S. (2021). Tuber and tuberous root development. Annu. Rev. Plant Biol. 72, 551–580. doi: 10.1146/annurev-arplant-080720-084456 33788583

