# Supplementary material for: A chromosome-scale genome assembly of turmeric provides insights into curcumin biosynthesis and tuber formation mechanism
Source: Front Plant Sci. 2022 Sep 26;13:1003835. doi: 10.3389/fpls.2022.1003835 (PMC9549246; doi:10.3389/fpls.2022.1003835)
Supplement: Supplementary file 1 [file DataSheet_1.docx]

**Table S1**. Summary of sequencing data generated in this study.

| **Library type** | **Platform** | **Reads number** | **Total base** | **Read N50** | **Coverage** |
| --- | --- | --- | --- | --- | --- |
| Long reads | PacBio Sequel | 8,768,425 | 224.90Gb | 35,284bp | 202x |
| Short reads | Illumina | 2 x 329,867,503 | 98.73Gb | 2 x 150bp | 89x |
| Hi-C | Illumina | 2 x  355,770,067 | 106.73Gb | 2 x 150bp | 96x |

**Table S2.** Statistics of assembled *C. longa* assembly

| **Items** | **Contig**  **length(bp)** | **Contig**  **number** | **Scaffold**  **length(bp)** | **Scaffold**  **number** |
| --- | --- | --- | --- | --- |
| Total | 1,110,057,677 | 680 | 1,110,115,577 | 101 |
| Max | 7,170,677 | - | 89,696,121 | - |
| N50 | 2,339,679 | 150 | 50,121,839 | 10 |
| N60 | 2,026,724 | 201 | 47,053,952 | 12 |
| N70 | 1,665,350 | 262 | 45,138,063 | 14 |
| N80 | 1,267,837 | 337 | 41,824,940 | 17 |
| N90 | 908,213 | 441 | 39,063,747 | 20 |

**Table S3** Summary of the assembled *C. longa* pseudo-chromosomes

| **Pseudomolecule** | **Contig Number** | **Length** |
| --- | --- | --- |
| chr1 | 56 | 89,696,121 |
| chr2 | 36 | 65,325,895 |
| chr3 | 43 | 63,714,752 |
| chr4 | 33 | 57,596,293 |
| chr5 | 27 | 57,296,084 |
| chr6 | 27 | 56,291,114 |
| chr7 | 30 | 55,220,454 |
| chr8 | 29 | 54,227,725 |
| chr9 | 30 | 52,746,678 |
| chr10 | 31 | 50,121,839 |
| chr11 | 34 | 47,110,039 |
| chr12 | 23 | 47,053,952 |
| chr13 | 23 | 45,719,903 |
| chr14 | 23 | 45,138,063 |
| chr15 | 26 | 44,213,428 |
| chr16 | 23 | 44,002,946 |
| chr17 | 32 | 41,824,940 |
| chr18 | 25 | 39,864,722 |
| chr19 | 18 | 39,213,493 |
| chr20 | 14 | 39,063,747 |
| chr21 | 17 | 33,022,550 |
| Total anchored | 600 | 1,068,464,738 |
| Unanchored | 80 | 41,650,839 |

**Table S4.** Genome completeness assessment of *C. longa* genome by BUSCO.

| Categories | Number | Percent (%) |
| --- | --- | --- |
| Complete BUSCOs | 1537 | 95.2 |
| Complete and single‑copy BUSCOs | 1136 | 70.4 |
| Complete and duplicated BUSCOs | 401 | 24.8 |
| Fragmented BUSCOs | 17 | 1.1 |
| Missing BUSCOs | 60 | 3.7 |

**Table S5.** The repetitive sequences of *C. longa*

| **Type** | **Number** | **Length(bp)** | **Rate (%)** |
| --- | --- | --- | --- |
| **Retroelements** | 641,706 | 676,957,358 | 60.98 |
| LINES | 12,869 | 7,136,748 | 0.64 |
| RTE/Bov-B | 11,562 | 6,523,285 | 0.59 |
| LI/CIN4 | 1,307 | 613,463 | 0.06 |
| LTR/Copia | 248,204 | 345,170,178 | 31.09 |
| LTR/Gypsy | 174,915 | 237,547,150 | 21.40 |
| **DNA transposons** | 353,942 | 93,254,340 | 8.40 |
| Hobo-Activator | 4,978 | 3,357,497 | 0.30 |
| Tourist/Harbinger | 2,134 | 1,173,122 | 0.11 |
| Rolling-circles | 1,065 | 917,584 | 0.08 |
| SSR | 94 | 5,406 | 0.01 |
| Unknown | 36,474 | 5,784,303 | 0.52 |
| **Total** | 1,033,281 | 77,6918,991 | 69.99 |

**Table S6.** Gene functional annotation of *C. longa* based on different database.

| Database | Annotated_Number |
| --- | --- |
| KEGG | 21,135 |
| KOG | 23,857 |
| Swissprot | 28,454 |
| TrEMBL | 48,501 |
| NR | 46,524 |
| All | 49,612 |

**Table S7.** Noncoding RNA prediction of *C. longa.*

| Type | Number |
| --- | --- |
| rRNA | 1,285 |
| tRNA | 1,829 |
| snRNA | 7,034 |
| sRNA | 111 |
| miRNA | 354 |

**Table S8. Gene family classification statistics**

| Species | Number of  genes | Number of genes in orthogroups | Number of orthogroups containing species | Number of species‑specific orthogroups | Number of genes in species‑specific orthogroups |
| --- | --- | --- | --- | --- | --- |
| *M. acuminate* | 36,979 | 34,221 | 16,375 | 173 | 753 |
| *M. balbisiana* | 33,021 | 30,051 | 15,585 | 167 | 580 |
| *Z. mays* | 34,337 | 32,047 | 14,665 | 930 | 3,897 |
| *A. thaliana* | 27,562 | 24,896 | 12,676 | 1,085 | 5,189 |
| *E. glaucum* | 36,836 | 30,701 | 14,809 | 215 | 606 |
| *C. longa* | 60,686 | 52,499 | 16,835 | 5,222 | 28,253 |
| *Z. officinale* | 73,003 | 68,373 | 17,073 | 3,195 | 13,946 |
| *O. sativa* | 39,133 | 32,188 | 15,136 | 1,519 | 7,154 |
| *P. dactylifera* | 29,239 | 28,353 | 13,277 | 357 | 1,490 |


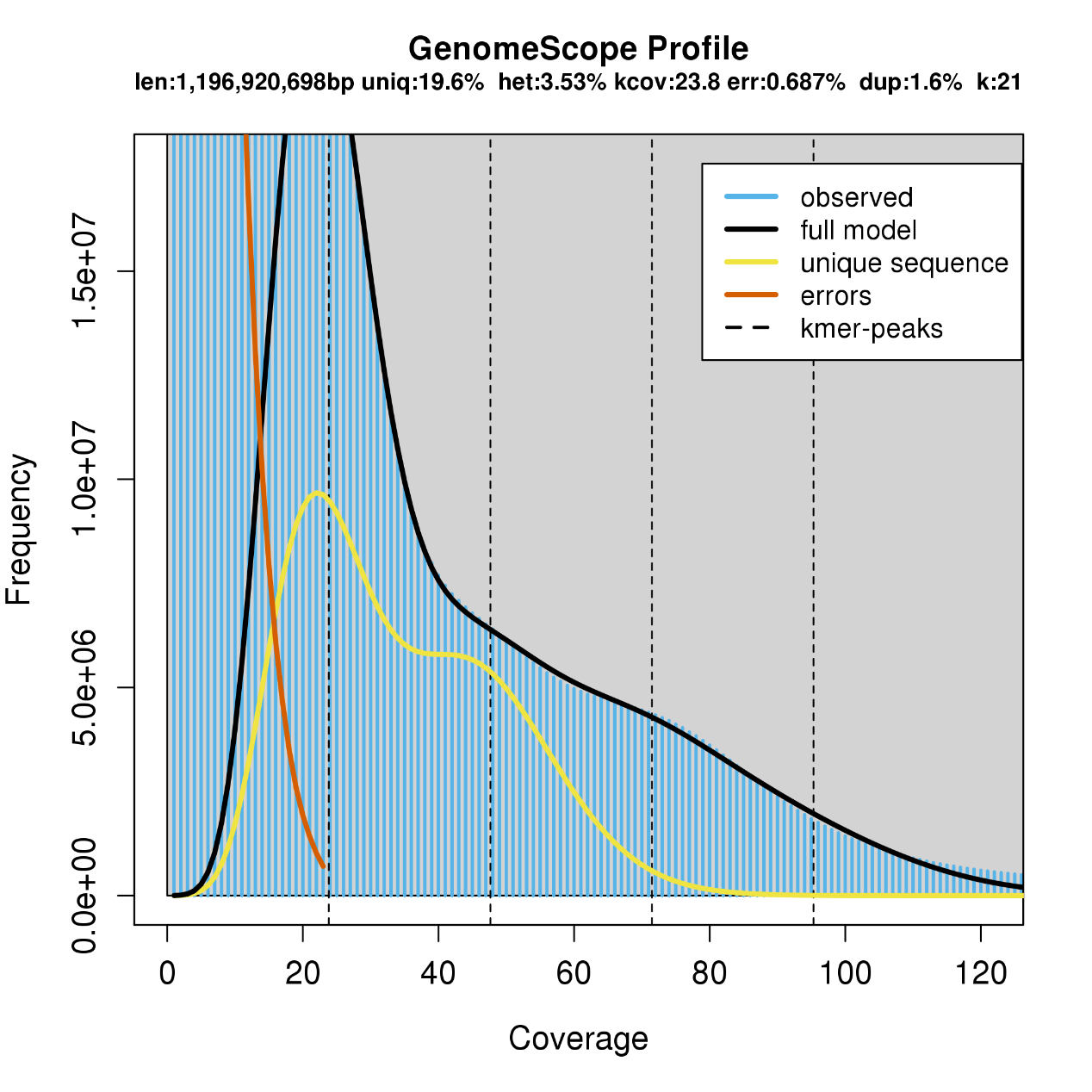


**Supplementary Information** **Fig. S1** K‑mer frequency distribution curve of the turmeric genome by GenomeScope. The turmeric genome was estimated to be ~1.19Gb with 3.53% heterozygosity at 21-mer.


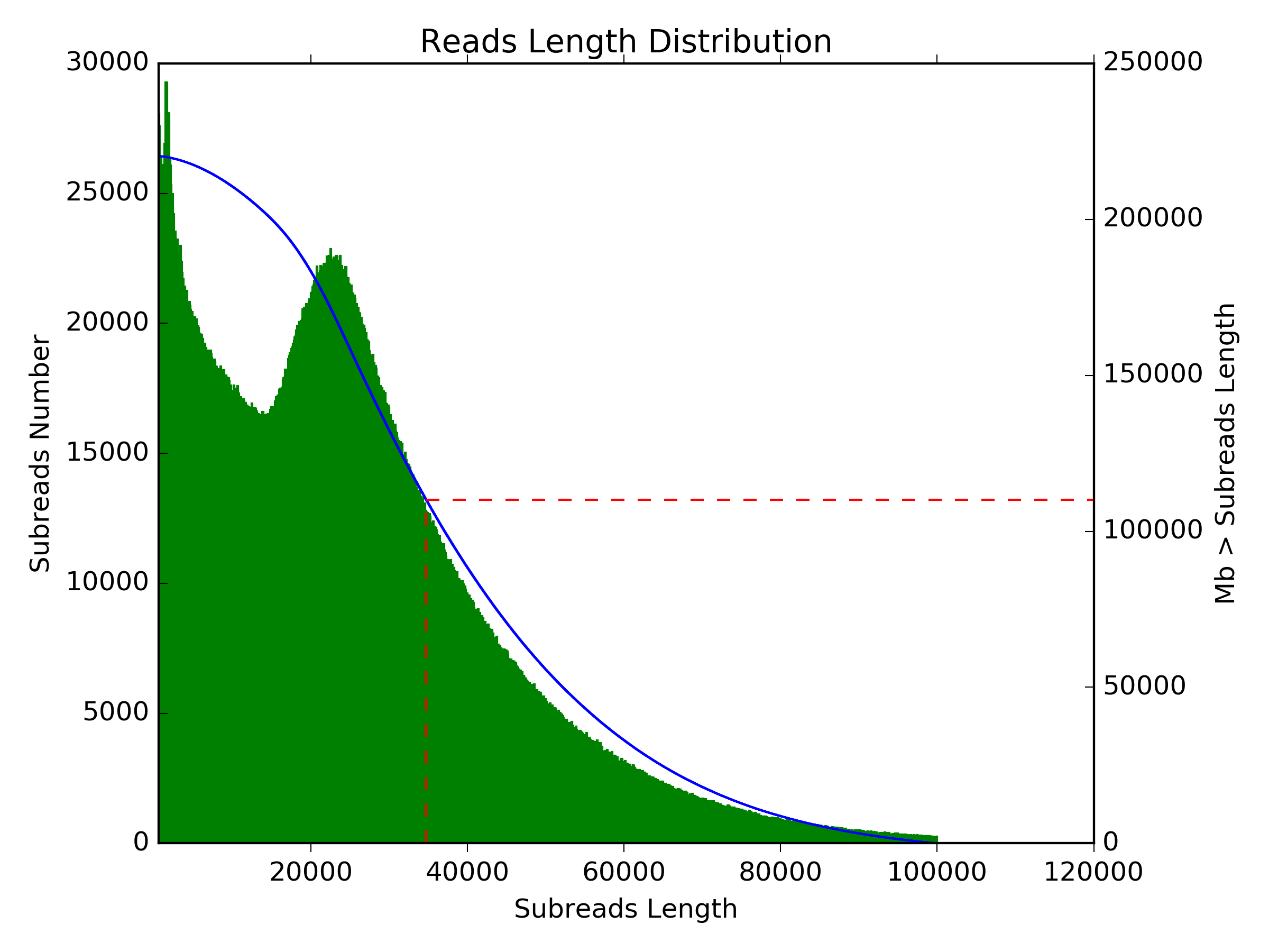


**Supplementary Information Fig. S2** Length distribution of subreads obtained from PacBio sequencing.


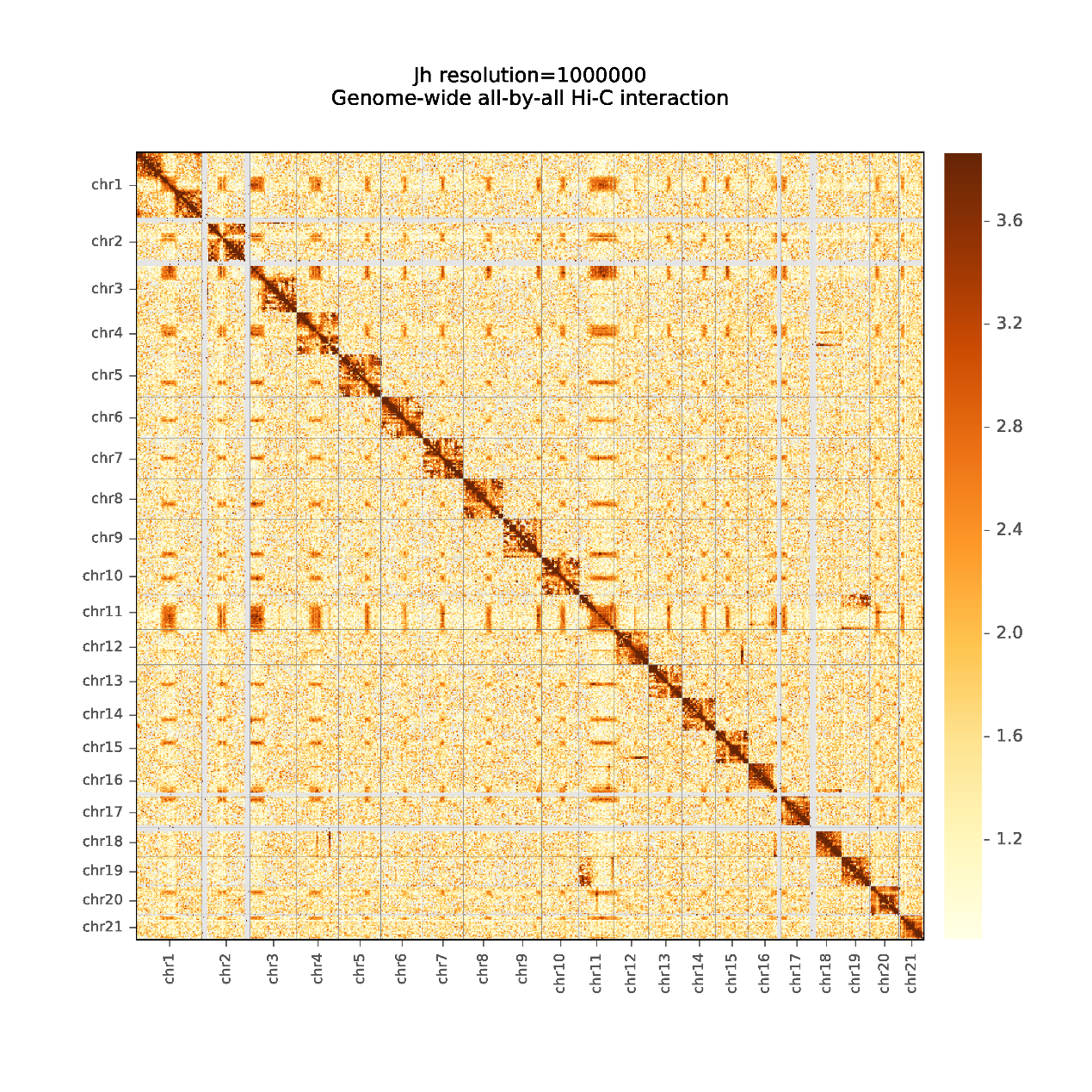


**Supplementary Information Fig. S3** The Hi-C map of turmeric genome assembly.


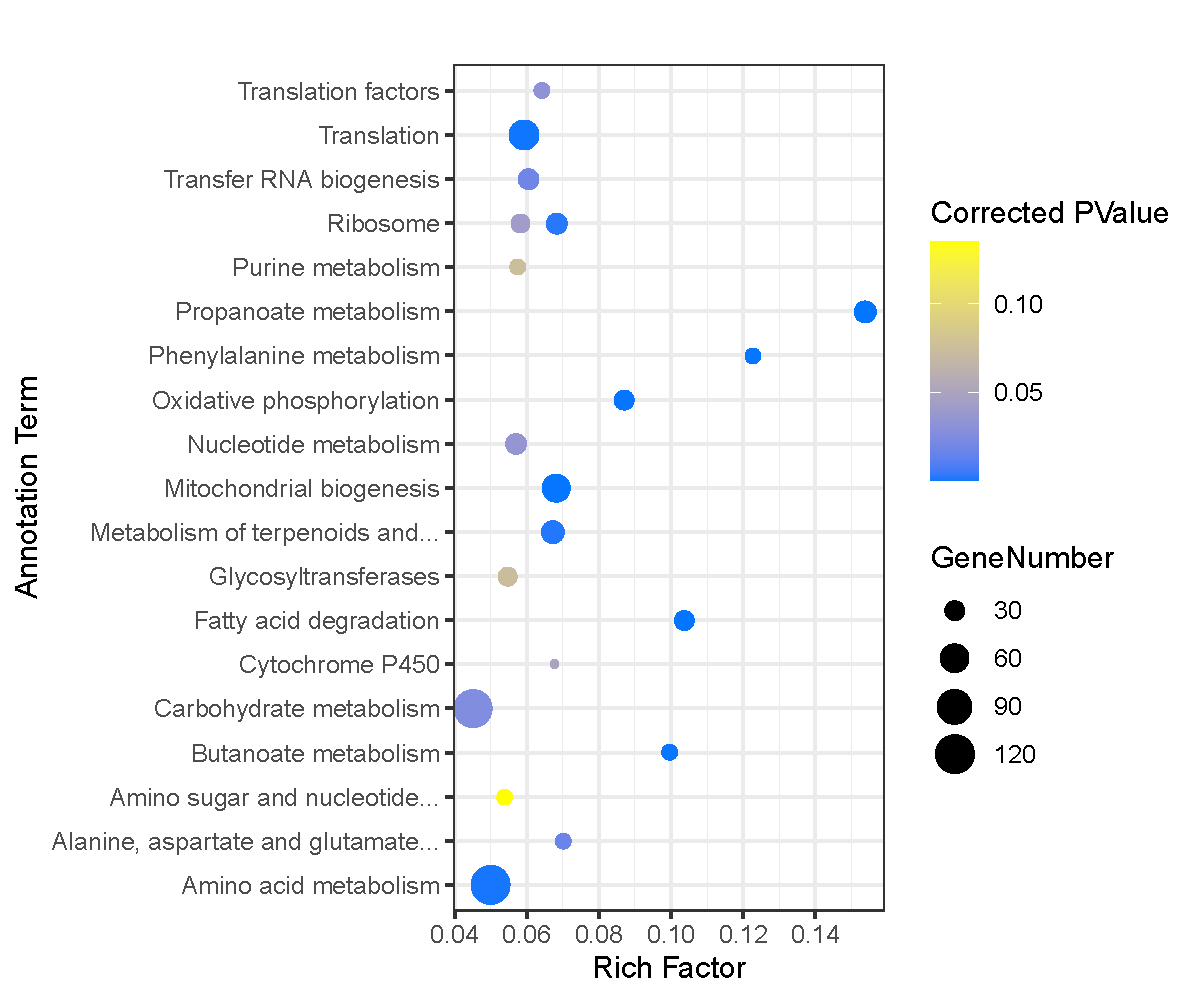


**Supplementary Information Fig. S4** KEGG enrichment analysis of genes in the expansion of gene families in *Curcuma longa*.


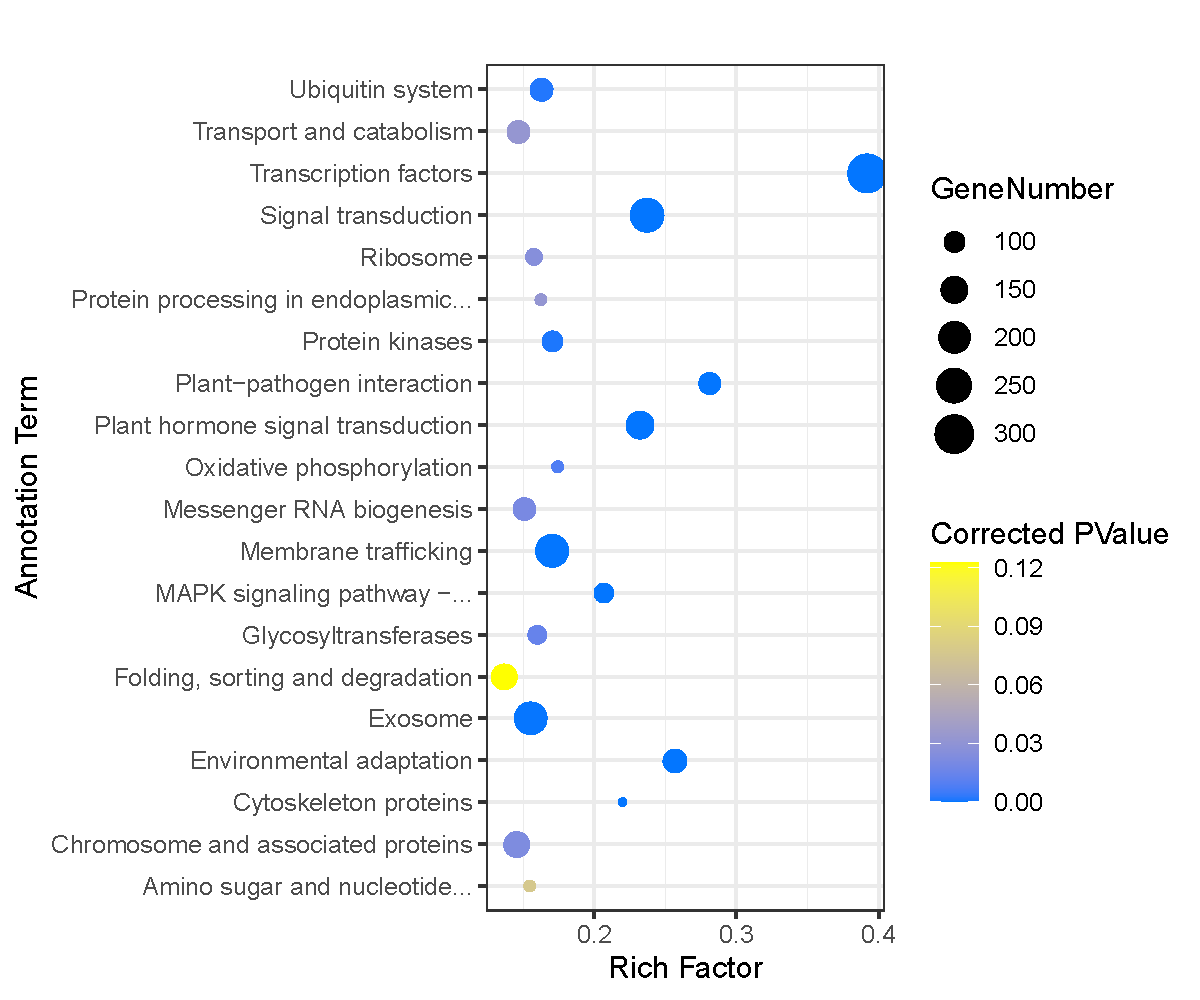


**Supplementary Information Fig. S5** KEGG enrichment analysis of genes in the contraction of gene families in *Curcuma longa*.


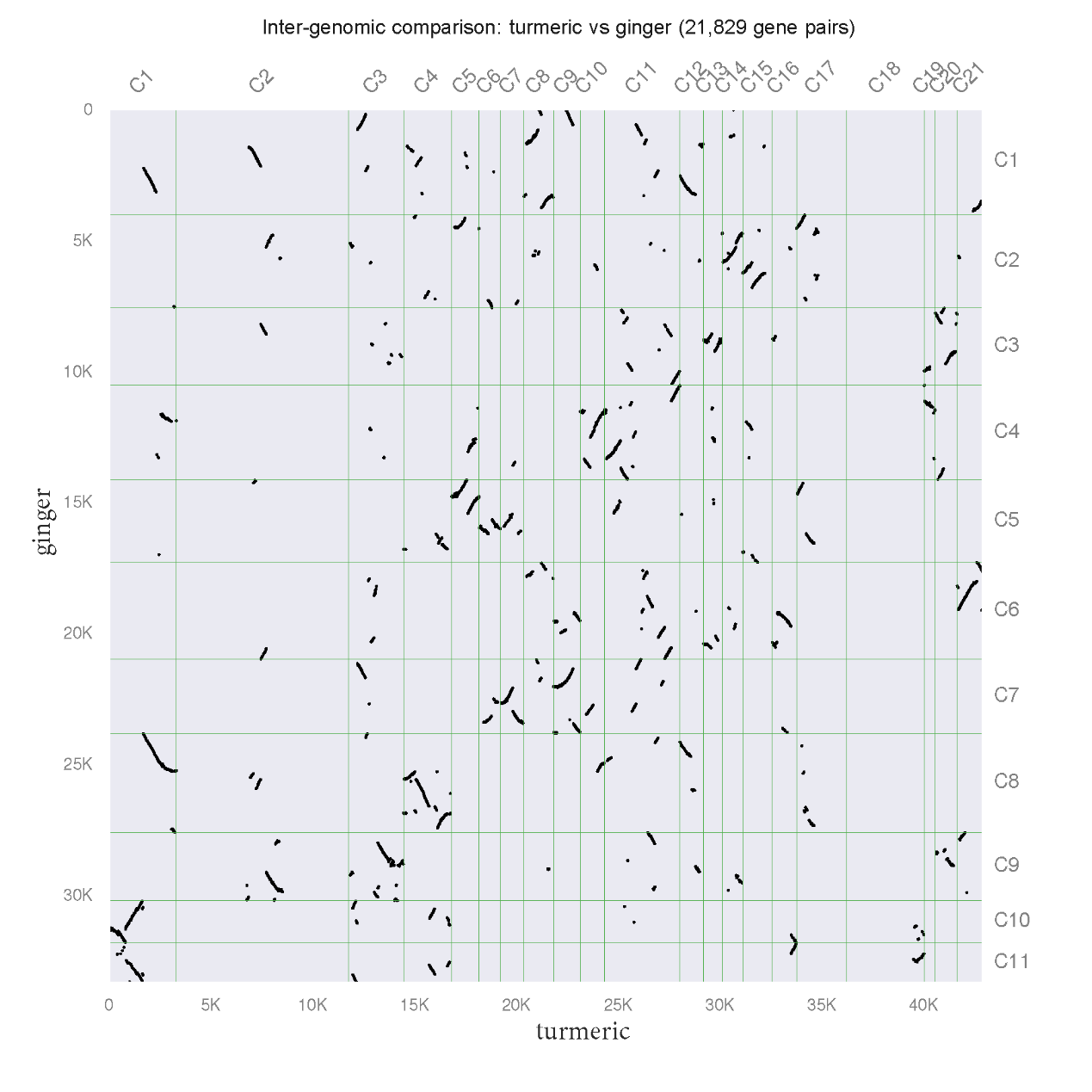


**Supplementary Information Fig. S6** Syntenic dot plot illustrating the comparative analysis of the turmeric and ginger genomes.


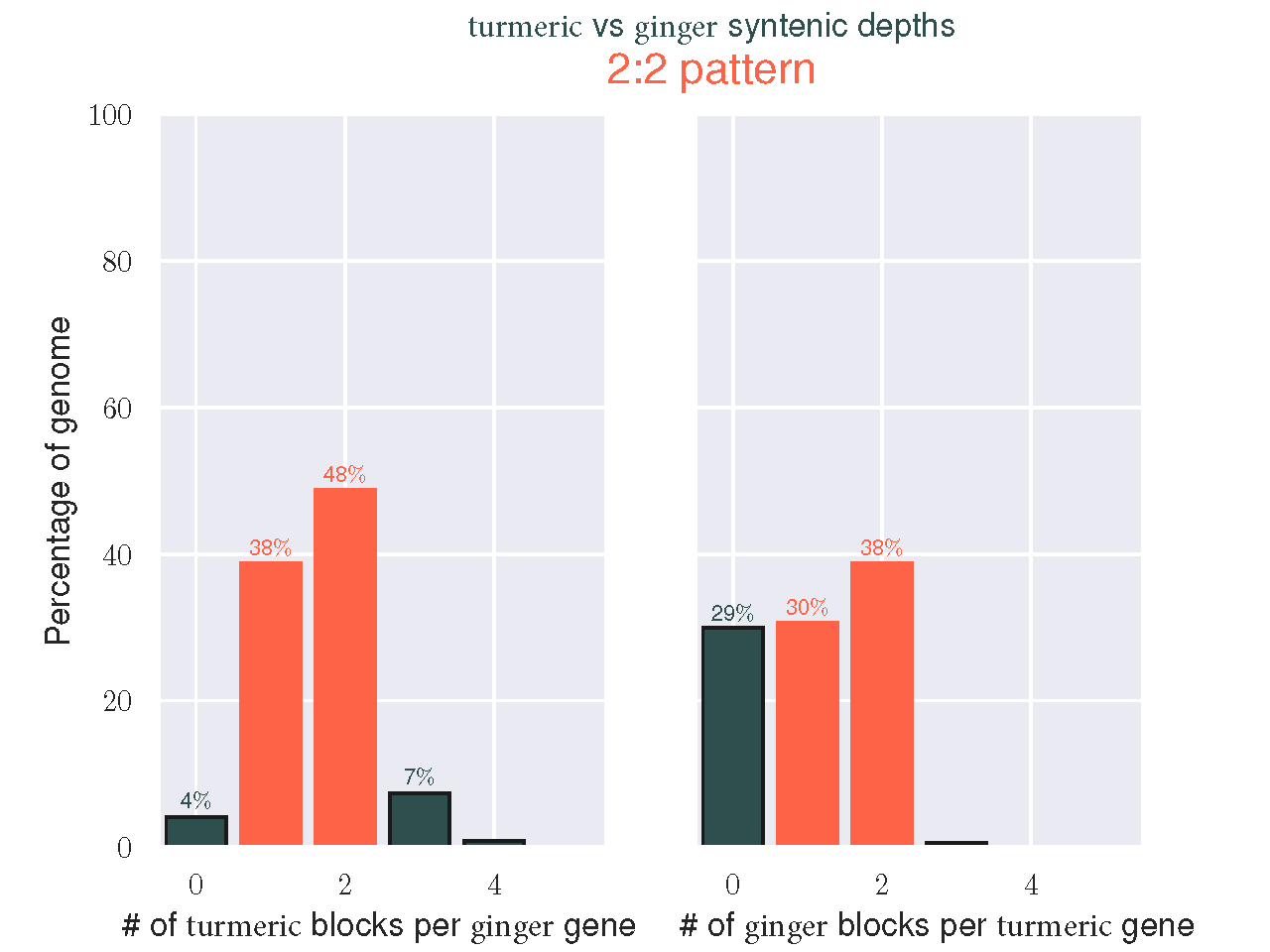


**Supplementary Information Fig. S7** The syntenic depth ratio between the turmeric and ginger genomes.


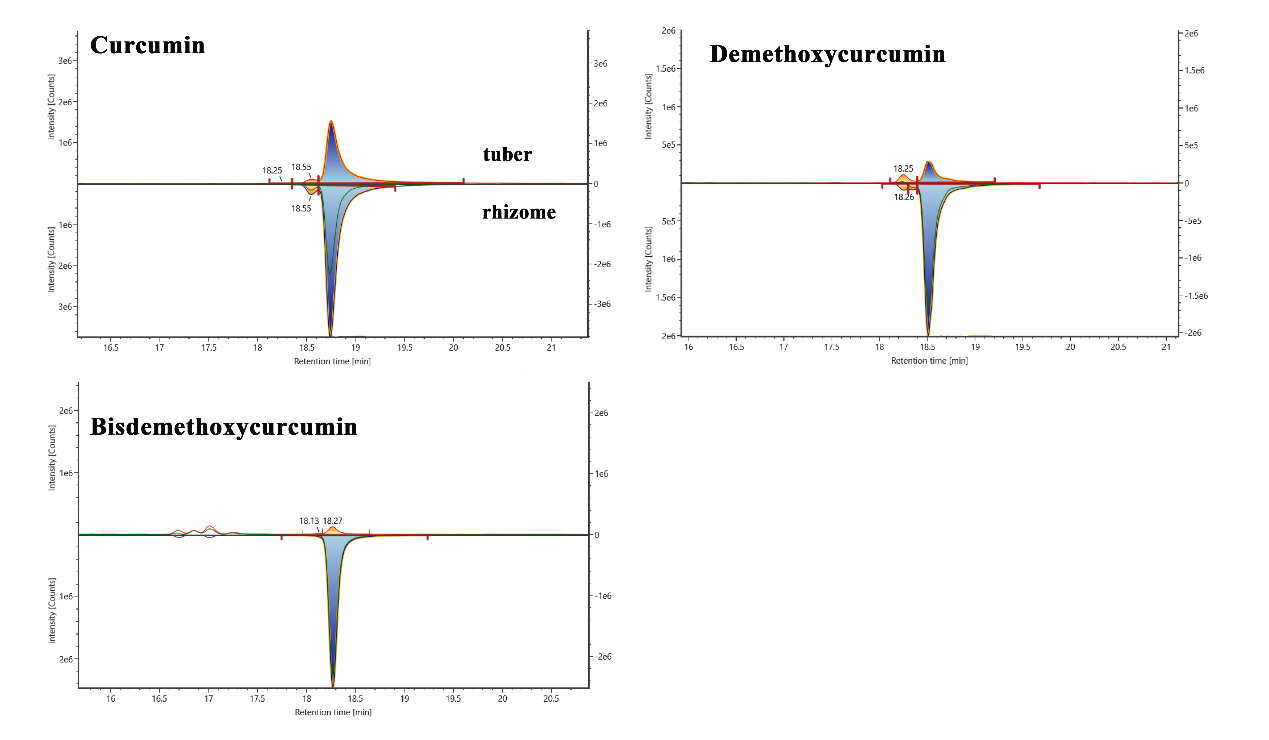


**Supplementary Information Fig. S8** Comparison of UPLC-MS peaks of three curcumin compounds in the rhizomes and tubers of *Curcuma longa*.


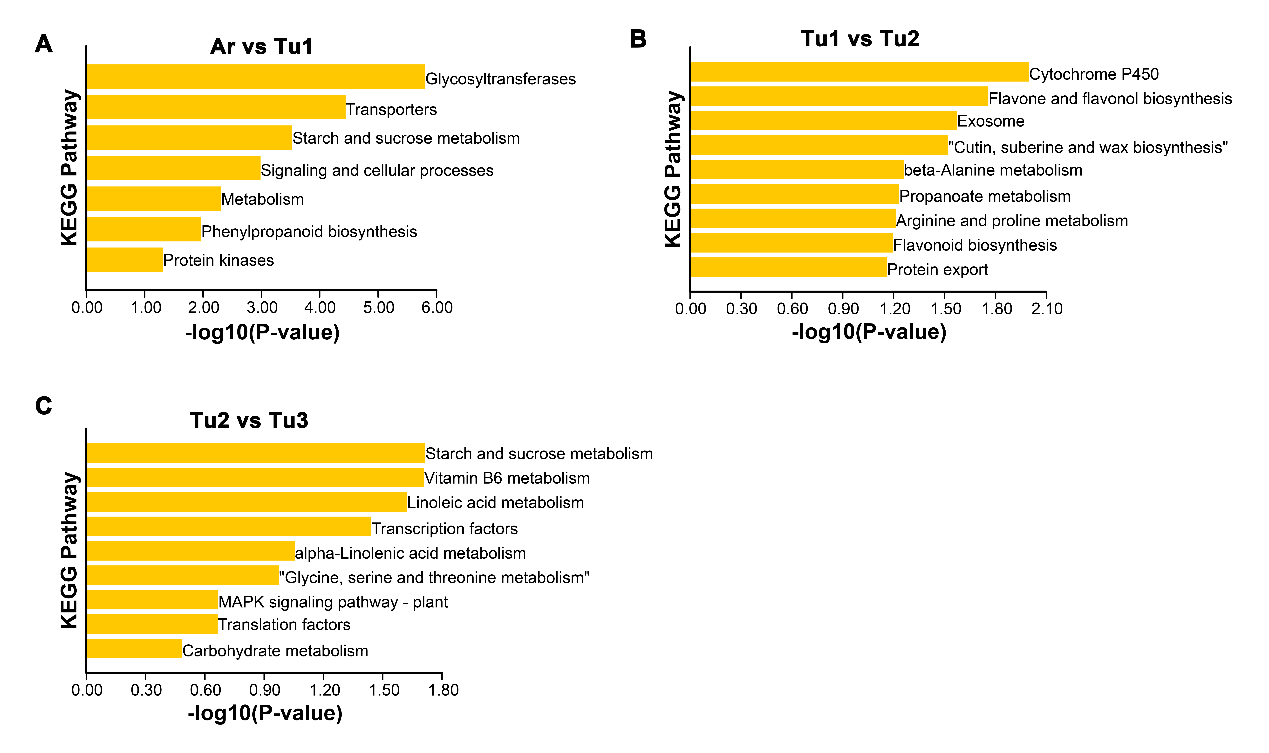


**Supplementary Information Fig. S9** KEGG enrichment analysis of DEGs in two comparisons, **A** Ar vs Tu1, **B** Tu1 vs Tu2, **C** Tu2 vs Tu3.
